# Hybrid diacrylate resin-gelatin methacryloyl composite with bone-to-brain stiffness range

**DOI:** 10.1038/s43246-025-00931-y

**Published:** 2025-10-02

**Authors:** Mohammad Naghavi Zadeh, Kapil D. Patel, Daniel Gosden, James A. Smith, Paul J. Gates, Qiukai Qi, Fabrizio Scarpa, Andrew Conn, Adam W. Perriman, Jonathan Rossiter

**Affiliations:** 1 School of Engineering Mathematics and Technology, University of Bristol, Bristol, UK; 2 School of Cellular and Molecular Medicine, University of Bristol, Bristol, UK; 3 John Curtin School of Medical Research, Australian National University, Canberra, ACT Australia; 4 Research School of Chemistry, Australian National University, Canberra, ACT Australia; 5 School of Chemistry, University of Bristol, Bristol, UK; 6 Bristol Composites Institute, University of Bristol, Bristol, UK

**Keywords:** Soft materials, Bioinspired materials

## Abstract

Biointerfacing techniques for connecting implants to living tissues are advancing, but matching stiffness at hard-soft interfaces, such as between tendon and bone, remains challenging. This is critical for improving biomechanical tissue models, repairing trauma, and integrating soft robotic technologies like artificial muscles. Here we introduce a 3D-printable, biocompatible composite combining a hydrogel (gelatin methacryloyl) with a hybrid resin of diacrylates and epoxide. By adjusting the mixture ratio, the material’s elastic modulus spans a wide physiological range, from 15 kPa (soft brain tissue) to 1.4 GPa (similar to bone), covering six orders of magnitude. Mechanical tests confirm this tunability, and cytocompatibility tests show high cell viability, proliferation, and metabolic activity. The approach offers a path to creating efficient gradient stiffness interfaces, potentially leading to more accurate tissue phantoms and devices for human body repair and augmentation, especially where continuous hard-to-soft transitions are essential.

## Abstract (web summary)

Biointerfacing techniques face challenges in stiffness matching at interfaces between hard and soft tissues, crucial for advanced biomechanical applications. Here, a 3D printable, biocompatible composite is developed that achieves a wide range of elastic moduli from 15 kPa to 1.4 GPa.

## Introduction

The mismatch of mechanical properties at the interfaces between two different materials is an intrinsic source of disbonding and crack propagation due to stress concentrations that emerge at the material’s singularity^1^. In nature, complex biological functionally graded materials (Bio-FGM) have evolved that can handle differences through continuous transitions, which solve the problem of spatial property mismatch, especially at the interfaces^2,3^. Bio-FGMs that tune the transition of elastic properties, and especially the elastic modulus, are well-known at interfaces such as dentin-enamel, enamel-cementum, ligament-bone and tendon-bone entheses^4,5^. Inspired by these examples in nature, man-made gradient interfaces^6^ are found in applications related to wound healing^7^, soft-hard mechanical^8^ and conductive interfaces^9^, and in the functionalized surface of orthopedic implants^10,11^. The methodology to make the gradient structure and the choice of constitutive materials, such as hydrogels and composites, determine the values and range of elastic properties. These methodologies could be based on different methods, for example, 3D printing while implementing complementary techniques such as grayscale light exposure^12–16^ or using bubbles and sacrificial materials to create porous scaffolds (porogens) with gradient pore size and stiffness that is tuned for cell culture and differentiation^17–22^. Despite the evident versatility and biocompatibility of both hydrogels and heterogeneous composites, there are still challenges regarding the stiffness and fatigue life of hydrogels and the realization of a materials system that fills the critical gap between the kPa stiffness of soft tissue and the GPa stiffness of bone. A composite materials system that could replicate this stiffness range in biocompatible and readily fabricable materials would enable the development of more accurate tissue phantoms and more effective interfaces between natural tissues and man-made implants. In this paper, we focus on tuning the stiffness over a broad range of biological matter to bridge this gap.

To make a soft-hard gradient, the chemistry and mechanical properties play a pivotal role. On the more compliant side, hydrogels are well-known for their biocompatibility and softness, although they can be tailored to be very stiff, tough, highly stretchable, and conductive^23–29^. Here, we employ a hydrogel, gelatin methacryloyl (GelMA)^30^, for softness to reach the low elastic modulus required for stiffness matching with soft tissue and materials. GelMA can be molded or 3D-printed to create complex geometries and micro-structures such as microgels and microspheres, and combined with other additives to form functional composite biomaterials^31–33^.

On the hard side, polymers that can bond with GelMA to create double/multiple co-/interpenetrating networks^34^ and have high mechanical performance are highly sought after. Poly(ethylene glycol) diacrylate (PEGDA) has been used in several studies to improve the mechanical properties of GelMA in terms of stiffness^35^ and degradation^36^. In addition, PEGDA is biocompatible and is used as a hydrogel or a photocurable composite with GelMA for cartilage/bone defect treatments^37–39^, which proves the suitability of diacrylate-based monomers and oligomers in bonding/networking with GelMA. Furthermore, the mechanical performance of diacrylates (radical-based curing) can be further improved by hybridization with epoxides (cationic curing)^40^.

Here, we hypothesized that a composite of GelMA with a hybrid resin of acrylates and epoxides could enable the tuning of mechanical properties over a broad range of elastic moduli by tuning their mixture ratio. The multiple ingredients within such hybrid resins require an optimization process to achieve high-end mechanical properties^41^. It is therefore reasonable to use a hybrid resin that includes PEGDA, has a verified mechanical performance and also possesses a similar curing process to GelMA. Here, we explored the mechanical properties and fabrication methods of the photocurable composite of GelMA and a hybrid resin and evaluated its biocompatibility and 3D printability. Furthermore, we designed and manufactured soft-to-hard functionally gradient structures and evaluated their hardness distributions to study the effectiveness of manufacturing protocols. The results highlight the possibility of achieving a continuous range of elastic modulus between 15 kPa and 1.4 GPa. This range of elastic modulus covers a broad range of biological tissues from very soft, close to the brain, to hard, such as ligaments and bone. This broad range of stiffness potentially surpasses other material solutions, such as fiber reinforced hydrogels^42,43^, composites of GelMA and PEGDA^35,44^ or other biocompatible materials such as glass^13^. In terms of manufacturing, resin/GelMA composites provide the possibility of using the mixture ratio as the single tuning parameter, which allows the facile creation of gradient structures or spatially tuned stiffness using direct ink writing 3D printing. The broad range of stiffness that resin/GelMA composite provides has the potential to surpass other manufacturing methods, such as grayscale printing^14,45^ or methods that use electric fields, magnetic fields or ultrasound to control the distribution of micro/nano particles and platelets^6^.

## Results and discussion

### Resin/GelMA composite

**Fig. 1:**
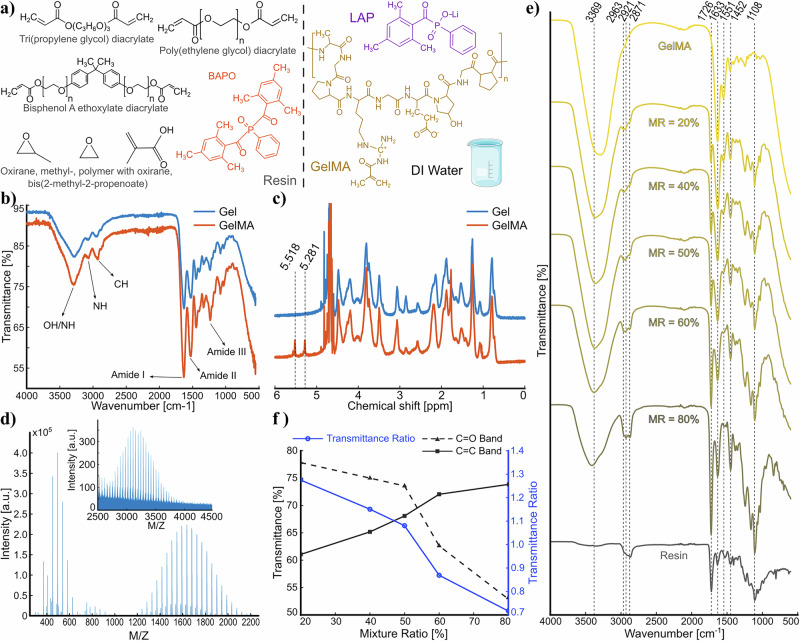
Chemical analysis. **a** Chemical composition of constitutive materials in resin/GelMA composite, **b** ATR-FTIR spectra of gelatin (Gel) before and after methacrylation (i.e., GelMA synthesis); **c** ^1^H-NMR spectra of Gel and GelMA confirm the substitution of primary amino groups by methacryloyl groups as peak signals at 5.281 and 5.518 ppm are assigned to methacrylate vinylic photons of methacrylic anhydride groups. **d** MALDI-TOF analysis of the liquid resin, where the inset shows zoomed view of the 2500–4000 M Z^−1^ band, **e** FTIR spectra of resin, GelMA, and resin/GelMA composites of different mixture ratios (MR), and **f** the trend observed between the transmission ratio and mixture ratio of the composite using C=C and C=O bands.

For the soft component, GelMA as synthesized was dissolved in deionized water at a temperature above 40 °C with 10% (w/v) concentration and 0.5% (w/v) Lithium phenyl-2,4,6-trimethylbenzoylphosphinate (LAP) for photoinitiation. For the hard component, we used a hybrid resin that combines PEGDA with epoxides, Tri(propylene glycol) diacrylate, Bisphenol A ethoxylate diacrylate, and BAPO as photoinitiator (Fig. 1a) and bonds with GelMA (Supplementary Information 1 and Supplementary Fig. 1). To create the composite, the mixture ratio (MR) was used to determine the volumetric ratio of the resin added to GelMA solution, where 0% (v/v) means pure GelMA solution and 100% (v/v) is pure resin. All composite mixture ratios mentioned in the text are volumetric (v/v).

The chemical characterization of synthesized GelMA was performed using ATR-FTIR, and ^1^H-NMR. The ATR-FTIR spectra of gelatin and GelMA (Fig. 1b) exhibited the presence of typical Amide bands of gelatin as Amide I, II, III, plus C–H and N-H stretching. The changes in peak vibrations amplitude between 1630 and 1660 cm^−1^ correspond to C=C and C=O bonds, which are associated with GelMA and the interaction between gelatin and methacrylic anhydride^46^, confirming the successful conversion of gelatin to GelMA. The ^1^H-NMR spectra of GelMA (Fig. 1c) exhibited two new peaks at 5.28 and 5.51 ppm, which belong to the methacryloyl groups of GelMA, confirming the methacrylation of gelatin, where degree of methacrylation was calculated as 80.25 ± 2.82%.

Mass spectroscopy (MALDI-TOF) was used to evaluate the mass distribution of PEG-based liquid resin and cured composite samples (Supplementary Fig. 22). Figure 1d demonstrates the mass distribution of monomers and oligomers in liquid resin in the pristine state, where a broad range of peaks are observed at low masses and a central peak at 1635 M Z^−1^. In addition, a minor peak (zoomed inset) with a center at 3109 M Z^−1^ is also present, and shorter spikes are distributed around both peaks with ~44 M Z^−1^ separation, indicating different numbers of ethylene oxide (oxirane) monomers in the chain. After cure, the mass distribution changes by polymerization, where peaks up to 10,000 M Z^−1^ in composite samples are found (Supplementary Information 4 and Supplementary Fig. 22). The average viscosity of the resin was ~180 mPa.s over a large range of strain rates (see Section 5), and the density was 1.15 g cm^−3^.

Figure 1e presents the FTIR spectrum of cured GelMA, resin, and their composites between 600 and 4000 cm^−1^. Two major peaks of cured GelMA belong to Amide I at 1634 cm^−1^ and Amide II at 1551 cm^−1^. The large band between ~3000–3700 cm^−1^ belongs to Amide A (N-H stretching), which is also affected by the O-H stretching due to the large water content in the composite. When resin is added, a peak at about 1726 cm^−1^ belonging to C=O stretching and a peak at 1633 cm^−1^ belonging to C=C bonds of acrylate also emerge, where C=C band overlaps with the strong Amide I of GelMA. When the mixture ratio increases (i.e., GelMA content reduces), the depth of bands related to GelMA also reduces. This is visually observed from the fading of the Amide II peak in the composite and reduction in depth of 1633 cm^−1^ band (Fig. 1e and C=C band in Fig. 1f) when the mixture ratio increases. For the C=O peak at 1726 cm^−1^ and C=C peak (that overlaps with Amide I) at 1633 cm^−1^, a trend between their transmittance ratio (C=O transmittance/C=C transmittance) and mixture ratio is observed (Fig. 1f), which suggests that FTIR can be used for evaluation of mixture ratio in this composite.

### Mechanical characterization

**Fig. 2:**
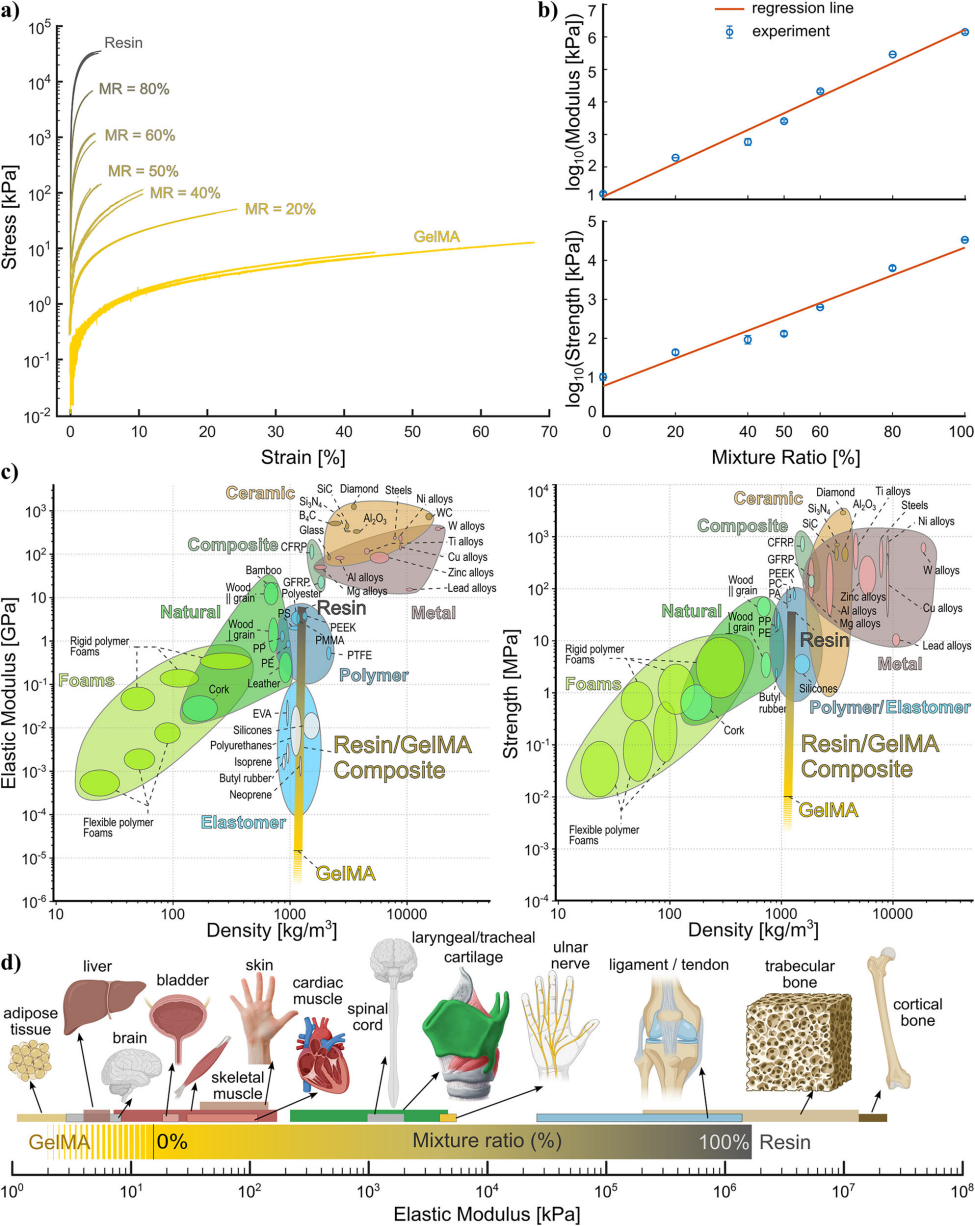
Mechanical performance. **a** Strain–stress curves of resin/GelMA samples with different Mixture ratios (MR), **b** logarithmic relationship of elastic modulus and strength with MR, **c** range of elastic modulus and strength covered by resin/GelMA composite comparing to other materials shown in Ashby plots (Chart created using data from CES EduPack 2019, ANSYS Granta^ⓒ^ 2020 Granta Design), and **d** comparison of elastic modulus for different mixture ratios of resin/GelMA composite with different biological tissues. Ranges of elastic moduli of tissues are approximate and obtained from refs. ^48–56^. Error bars represent standard deviations of three samples.

A minimum of three samples (Supplementary Table 1 and Supplementary Fig. 2) of GelMA, resin, and composites with mixture ratios of 20%, 40%, 50%, 60%, and 80% were tested and the overall stress-strain diagrams are presented in Fig. 2a. This number of points would be enough to capture the nonlinear behavior as a continuous trend between a minimum (GelMA) and maximum stiffness (resin). The FTIR analysis (Fig. 1e) also confirms the continuous variation in chemical bonding. While stiffness increases with mixture ratio, the ultimate strain capacity reduces. The elastic modulus using linear fit over a linear range (up to 2% strain) was obtained, and the values of elastic modulus and strength are presented in Table 1. Figure 2b demonstrates a linear correlation between the logarithm of the elastic modulus and strength and the mixture ratio. Coefficients for the logarithmic equations are presented in Table 2. Therefore, the mechanical properties of the composite can be predicted using the modulus-MR and strength-MR relationships as long as the moisture content does not change after curing. This composite material is suitable for moist environments, such as inside the body, where the GelMA part of the composite can retain its water content. The average modulus of the resin samples used in this study is ~1.42 GPa, while the softer component of GelMA with 10% w/v concentration has 15.0 kPa average modulus, which demonstrates the ability of this composite material to achieve arbitrary values between these two extremes, covering six orders of magnitude.

**Table 1:** Elastic properties for different mixture ratios

Mixture ratio (MR)	Mean Elastic modulus [kPa]	Mean strength [kPa]
GelMA	15.0 ± 1.4	10.4 ± 2.1
20%	192.1 ± 1.7	44.0 ± 6.4
40%	598.8 ± 136.7	94.0 ± 22.7
50%	(2.5763 ± 0.25) × 10^3^	131.9 ± 14.9
60%	(2.1336 ± 0.21) × 10^4^	628.4 ± 17.2
80%	(2.8893 ± 0.03) × 10^5^	(6.362 ± 0.75) × 10^3^
Resin	(1.4178 ± 0.13) × 10^6^	(3.6303 ± 0.12) × 10^4^

Resin/GelMA composite tensile test results presenting mean values ± standard deviation.

**Table 2:** Correlation between elastic properties and mixture ratio

Regression equation: *f*(*MR*) = 10^(*a*. *MR*+*b*)^			
Property *f*(*MR*)	*a*	*b*	regression error (*R*^2^)
Elastic Modulus [kPa]	0.0514	1.0850	0.9970
Strength [kPa]	0.0356	0.7732	0.9926

Regression relation and coefficients of elastic modulus and strength with respect to the mixture ratio.

To further illustrate the range of material categories that the resin/GelMA composite can match, Ashby plots of elastic modulus and strength for different materials are shown in Fig. 2c, d. If lower concentrations (<10% w/v) of GelMA were used, a lower modulus down to 1 kPa could be achieved^47^(stripe color in Fig. 2c, d), yielding a range of seven orders of magnitude in modulus. In terms of elastic modulus and strength, it is clear from Fig. 2c, d that the resin/GelMA composite can match most elastomers, polymers, natural materials, and rigid and flexible foams.

To better understand how the resin/GelMA composite provides stiffness matching organs and tissues, a comparative diagram is presented in Fig. 2d, where the approximate range of elastic moduli (expressed as kPa for clarity) of different organs and tissues reflects the corresponding mixture ratios of the resin/GelMA composite. On the more compliant end of the resin/GelMA composite (≈10 kPa), GelMA with 10% w/v concentration has an average modulus that provides stiffness matching with many soft tissues such as the bladder, skeletal and cardiac muscle, and skin^48^. Lower concentrations of GelMA can provide an elastic modulus down to 1 kPa^47^, which can match the stiffness of vocal folds^49^, brain, and liver tissues^48^. In the mid-range of elastic modulus, the nervous system (ulnar nerves and spinal cord)^48^ and tracheal and laryngeal cartilages are covered by the composite material (mixture ratio  ≈50%)^50,51^. On the stiff end of the composite, which is pure resin, its average elastic modulus of 1.4 GPa covers that of the trabecular bone and is close to the range of cortical bone (≈17.9 GPa)^52,53^. Moreover, the composite material also covers the elastic modulus for tendons and ligaments, where a broad range of 13 MPa to 1470 MPa is reported^54–56^.

### Biocompatibility study

**Fig. 3:**
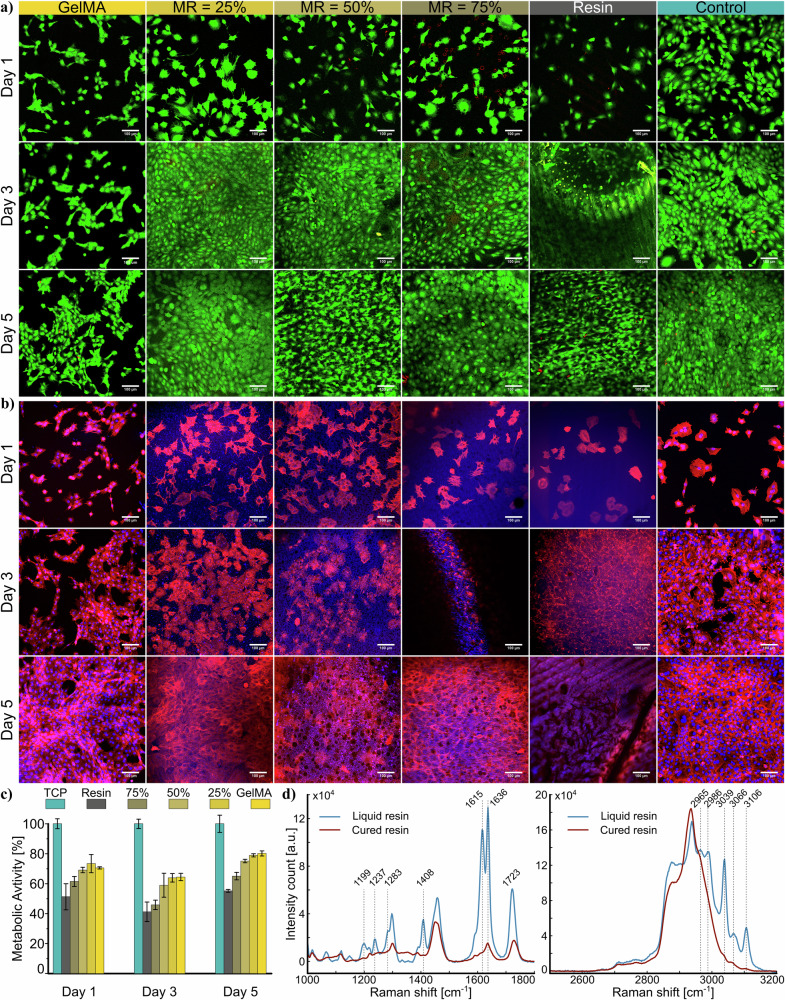
Biocompatibility analysis. Cytocompatibility study on different mixture ratios on day 1, 3, and 5 post culture: **a** cell viability analysis, **b** cell proliferation using DAPI/Phalloidin, **c** normalized metabolic activity from AlamarBlue analysis, and **d** Raman spectrum of liquid and cured resin. Scale bars represent 100 microns.

As a biopolymer, GelMA has excellent biological compatibility and is a potential candidate for tissue engineering applications^46^. It is therefore important to understand how modification affects its biocompatibility. To investigate the influence of the resin in resin/GelMA composite on cell viability and cell proliferation, C2C12 cells were seeded and cultured in vitro for days 1, 3, and 5. To specifically evaluate cell proliferation, we employed the AlamarBlue assay, a non-destructive method to assess cell metabolic activity, which is commonly used as an indirect indicator of cell viability and proliferation. The LIVE/DEAD staining fluorescence images of C2C12 cells on resin/GelMA samples for day 1 exhibited higher cell adhesion and viability (green; calcein AM) on GelMA and resin/GelMA samples compared to resin only (Fig. 3a). Moreover, cell numbers increased at day 3 and day 5 on all composite and pure resin samples, which confirmed high cell viability (further details in Supplementary Information 3 and Supplementary Figs. 7–18). A few dead cells (red; propidium iodide) were visible on the resin samples, possibly due to the leaching of unreacted monomers of resin or photoinitiator. Therefore, ensuring complete crosslinking and thorough washing of the resin/GelMA composites is critical to mitigate such risks. Over time, GelMA hydrogel may degrade enzymatically or hydrolytically, releasing oligopeptides, methacrylic acid derivatives, or resin byproducts^57,58^. However, these degradation products have not shown significant cytotoxic effects. Based on our cellular activity data, we do not observe any potential long-term cytotoxicity from the resin/GelMA samples, provided that the samples are properly photo-crosslinked and rigorously washed. Overall, LIVE/DEAD staining results confirmed that resin/GelMA composite samples have cell viability and also confirmed that properly washed resin samples endowed long-term cell viability. The confocal images confirmed that the C2C12 cells were predominantly alive and proliferated with time.

**Fig. 4:**
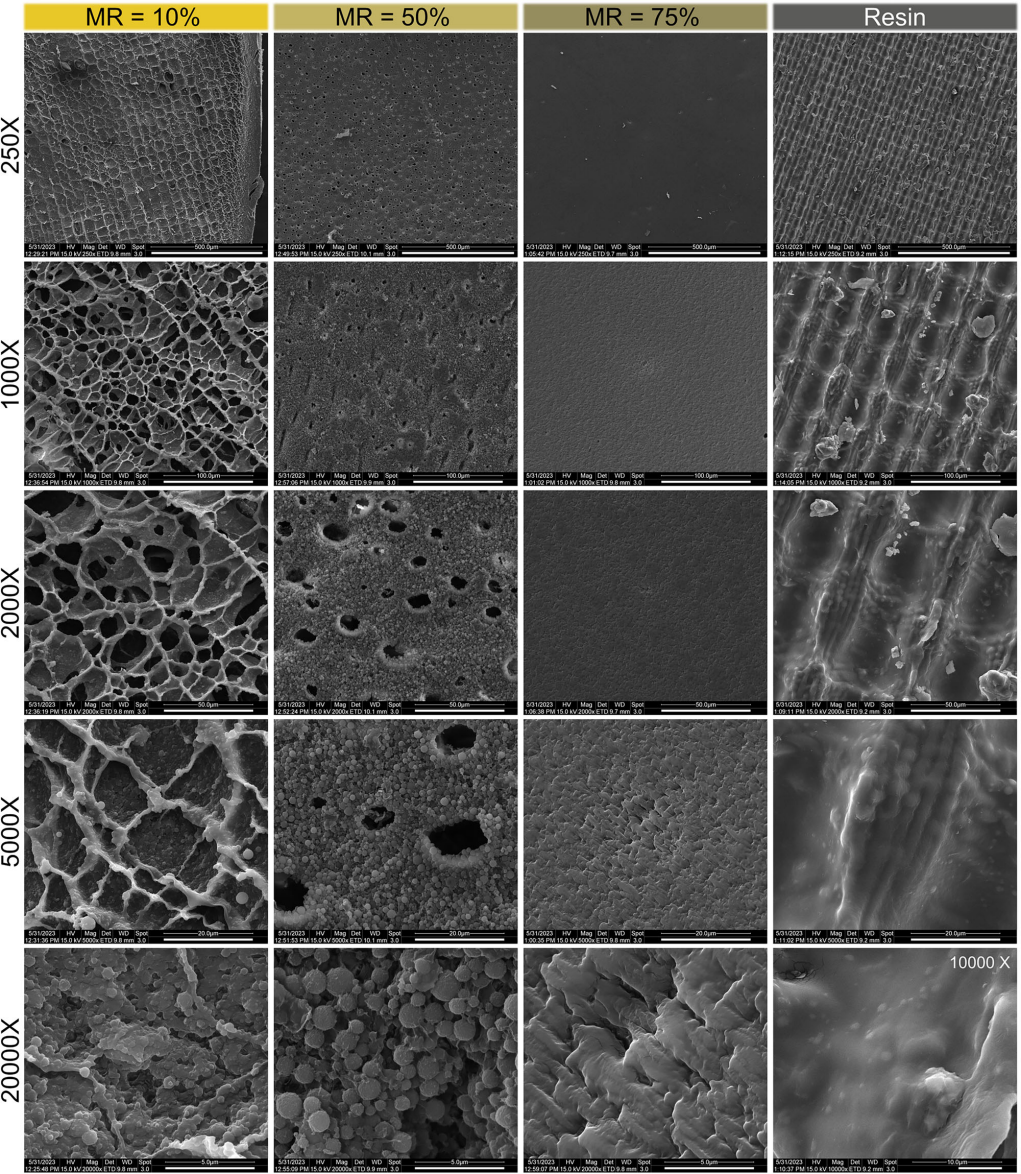
SEM evaluation. FE-SEM images of the resin and composite material with mixture ratios of 10%, 50%, and 75% magnification factors (scale bar represents) of ×250 (500 μm), ×1000 (100 μm), ×2000 (50 μm), ×5000 (20 μm), and ×20,000 (5 μm).

For cell proliferation study, the cells were stained with 4,6-diamidino-2-phenylindole (DAPI, blue; nucleus), and phalloidin (red; cytoskeleton), where fluorescence images of cells adhered on composite samples exhibited mostly spread morphology (Fig. 3b). Day 5 showed proliferation on all samples, while GelMA, MR = 25%, MR = 50%, and MR = 75% samples exhibited higher cell proliferation compared to pure resin. The increase in cell proliferation and spreading can be attributed in part to the proportion of GelMA in the composite, which introduces bioactive motifs (such as RGD sites) that promote cell adhesion. Additionally, the mechanical stiffness of the material plays a crucial role in regulating cellular behavior through mechanotransduction pathways. Furthermore, the surface morphology observed in SEM images provides topographical cues that support cell attachment, spreading, and cytoskeletal organization (Fig. 4).

The cellular metabolic activity results from AlamarBlue assay (Fig. 3c and Supplementary Information 3 and Supplementary Figs. 19–20) show that over Day 1, Day 3, and Day 5 on different samples with varying compositions (TCP, 100%, 75%, 50%, 25%, 0%). TCP result has been considered as control, and among hydrogel samples, higher GelMA content (100%, 75%) supports better metabolic activity than lower percentages (50%, 25%, 0%). Statistically, as shown in Supplementary Fig. 20, significant differences (*p < 0.05) are observed between multiple groups, especially between TCP and lower GelMA content groups. Overall, GelMA content correlates with improved cell metabolic activity, suggesting enhanced biocompatibility and support for cell proliferation over time.

The surface morphology of the samples affects cell adhesion and proliferation. Therefore, SEM analysis was used to evaluate 3D-printed resin samples and composites of 10%, 50%, and 75% resin content at different magnification factors (Fig. 4). GelMA is well known to have a porous structure with open pores, while adding PEGDA reduces the pore size and increases the wall thickness of the pores^35,38^. In resin/GelMA composites, the porous structure of the GelMA with microscale open pores was visible (×1000 and ×2000) for low mixture ratios (10%), while a secondary scaffold-shaped network from the resin was also formed. By increasing the mixture ratio to 50%, the co-network of resin and GelMA was affected by the water solubility that results from local hydrophobic/hydrophilic interactions, causing microsphere formation in the PEG-based resin^59^. When the mixture ratio increases to 75%, the resin-based network dominates, and small-scale roughness (asperities smaller than 5 μm) is observed over the surface. The pure resin samples were 3D printed to facilitate the UV light exposure of successive thin films of resin to minimize the uncured monomer content, which causes the visible surface pattern. At a high magnification (×10,000), the surface of the cured resin is observed to be much smoother than the composite samples. This change of microstructure and porosity not only affects the cell growth, differentiation, and alignment but also the microscale deformations and overall stiffness and mechanical properties.

GelMA and LAP are known materials in terms of cytocompatibility, and hence toxins in this composite originate from the resin^60,61^. As a rapid and non-contact method, Raman analysis was performed on the liquid resin sample and its cured and washed counterpart using green laser excitation (Fig. 3d and Supplementary Information 4 and Supplementary Fig. 21). Two peaks at 1615 and 1636 cm^−1^ belong to the C=C bonds that indicate the presence of acrylate groups in the liquid resin, which diminish considerably after cure as an indicator that acrylate-based monomers were polymerized. The presence of olefinic symmetric and asymmetric CH vibrations in sharp peaks between 3000-3100 cm^−1^ in liquid resin is also a clear indication of acrylates^62^, while oxirane also has peaks in this band^63,64^. These various peak indicators in the Raman spectrum of the liquid versus cured resin showed that Raman spectroscopy can be used as a rapid, non-contact method to evaluate the degree of cure and the presence of toxins in the samples.

### Gradient structure

Considering the biocompatibility of the resin/GelMA composite and its broad range of elastic modulus, the development of the composite gradient interfaces (CGI) is now explored. In terms of potential applications using CGI, stiffness matching between the artificial muscles and the target anchor (bone, tendon or cartilage) enables load-bearing performance and life cycle improvement by mitigating interface stresses and failures due to elastic modulus mismatch. Moreover, soft implantable electrode arrays^65,66^, such as soft neural and brain interface devices that require transcutaneous implants and attachment to the skull, can also benefit from the CGI.

**Fig. 5:**
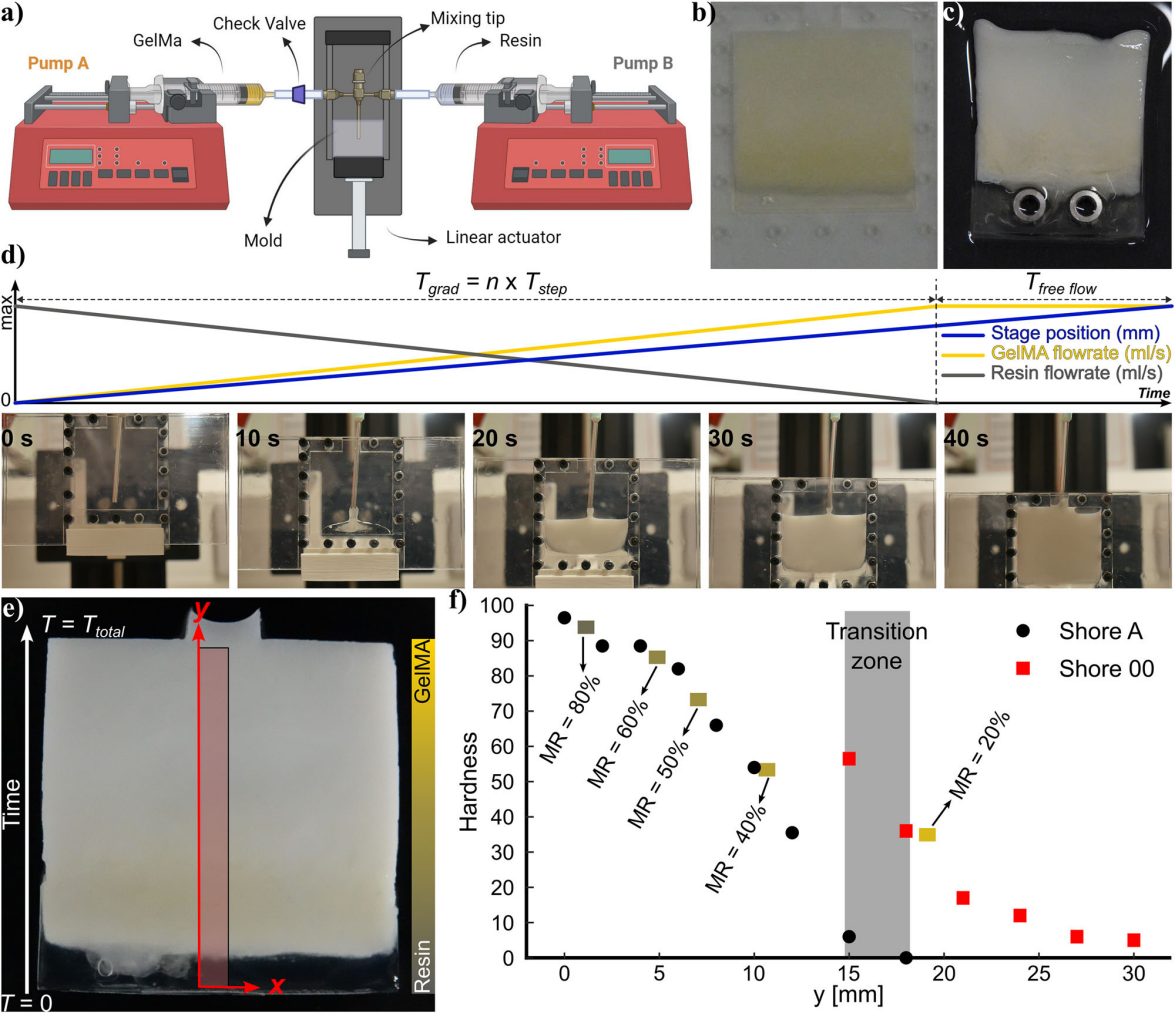
Gradient manufacturing and analysis. **a** The setup for manufacturing soft-hard gradient samples, **b** the gradient sample in the opened mold immediately after cure presents a gradient in yellow color from resin, **c** possibility of implementing in-mold inserts for ease of installation of gradient interface, **d** linear function of the injection for resin and GelMA in gradient manufacturing and snapshots from different sequences of the injection, **e** gradient sample used for the hardness measurements, **f** plot of the hardness in Shore-A and Shore-00 along the gradient direction of the sample where approximate position of different mixture ratios are highlighted.

Several approaches for manufacturing gradient biomaterials have previously been demonstrated, including active mixing and deposition, microfluidics, and buoyancy^67,68^ and voxel-based inkjet printing^8,69^, but they all have limits with respect to viscosity, photocuring, etc. For this work, we used a flow rate control approach with passive mixing and molding (as shown in Fig. 5a and Supplementary Fig. 23) to make linearly functionally graded resin/GelMA samples. After molding and curing, the gradient of the yellow resin color can be observed, suggesting a gradient distribution of resin in the sample (Fig. 5b). In addition to simple molds, metallic (or other rigid) inserts can be added to the mold at the resin-rich side of the interface to enhance connectivity and ease of use of the CGI when bolts or other standard fasteners are used (Fig. 5c).

We used linear flow rate functions with opposite slope values for GelMA and resin to make linear FGM samples (Fig. 5d). The resin started with high flow which decreased over the course of the injection, while GelMA started with no flow and reached its maximum flow rate at the end of injection. A constant injection time for GelMA was used to compensate for the volume of dead-space in the piping and static mixer. Images of the mold at different times of injection are shown in Fig. 5d.

The sample is resin-rich and hard at the bottom where injection begins (*T* = 0), and becomes very soft at the top where the injection ends (*T* = *T*_*total*_)(Fig. 5e). There was still a small amount of resin mixed with GelMA at the end due to the remnants in the mixing tip, resulting in the white color. In future work, the use of a micro-liter chamber and active mixing to improve the mixing dynamics and spatial resolution of the mixture ratio between resin and GelMA will be considered.

Measuring the elastic modulus over a broad range of kPa to GPa in a small sample with continuous variation of stiffness is challenging; therefore, we used durometry to measure the hardness locally. Initially, the hardness of samples of pure resin, pure GelMA, and different mixture ratios were measured separately as discussed in Supplementary Information 2 and Supplementary Figs. 3–4, where resin’s hardness exceeded 100 Shore-A and GelMA was zero Shore-00, thus demonstrating a very broad range of hardness across the composite space. Using two durometers for Shore-A and Shore-00 scales, we then analyzed the hardness variation along a 30 mm strip of a graded stiffness sample along the injection (gradient) direction (highlighted central strip in Fig. 5e). Figure 5f shows the smooth hardness variation along the *y*-direction. The highest hardness at the resin-rich end of the gradient sample was 96.5 Shore-A and the lowest hardness was at the GelMA-rich end with a hardness of 5 Shore-00. As hardness (Supplementary Fig. 4) and elastic modulus are both available for the given mixture ratios, it is possible to map between the two. Consequently, the approximate position of the hardness corresponding to different mixture ratios (20,40,50,60,80%) are also highlighted in Fig. 5f. This demonstrates the potential to achieve extremely large-range and smoothly varying functionally graded composites. In addition to mechanical test methods to confirm the gradient property, it is possible to use the color cues as shown in Supplementary Information 6 (Supplementary Figs. 24–25).

**Fig. 6:**
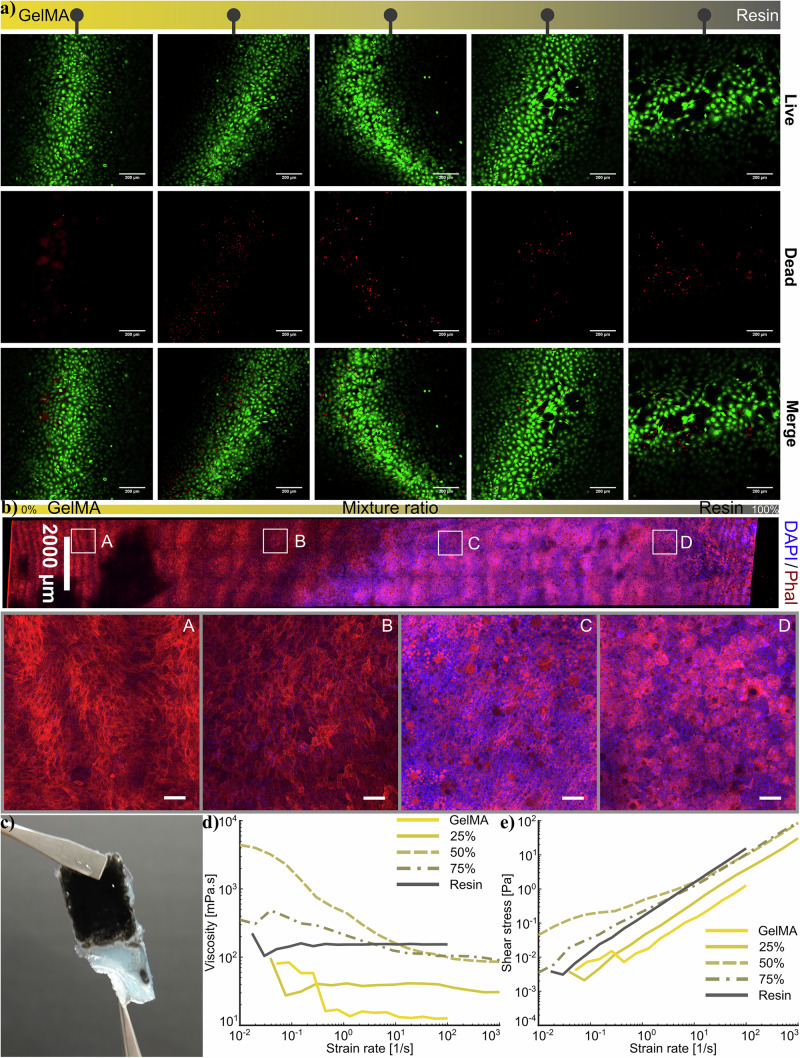
Gradient biocompatibility analysis and 3D printing investigation. **a** Cell viability analysis on Day 1 after culture (Scale bars present 200 microns) and, **b** cell morphology at different locations of the gradient sample from soft to hard, illustrated on Day 6 using DAPI/Phalloidin staining analysis (Scale bars present 100 microns). 3D printability of the resin/GelMA composite: **c** a 3D-printed sample using direct ink writing (resin with black pigments is used for better visibility) has been twisted between two tweezers, **d** variation in viscosity, and **e** shear stress with strain rate for resin/GelMA composite with different mixture ratios before cure.

The C2C12 cell viability on CGI samples in-term of LIVE/DEAD staining showed excellent results at day 1 (Fig. 6a) with higher number of cells at the softer side (GelMA) compared to the stiffer side (resin)(Supplementary Video 1). Furthermore, cell proliferation on CGI samples in terms of DAPI/phalloidin staining results for day 5 showed cell proliferation at all locations of the CGI sample (Fig. 6b), proving the CGI sample is biocompatible. Insets A, B, C, and D in Fig. 6b highlight the lower number of cell proliferation on the stiff side than the soft side. This may be due to the high stiffness effect and fewer matrix tethering points^70,71^; it has been reported that stiffness and surface morphology of composite materials play an important role in cell spreading and proliferation^72,73^. The DAPI/phalloidin staining results confirmed that the fabricated resin/GelMA composite and CGI samples are suitable for cell proliferation.

### Additive manufacturing

The possibility of 3D printing soft-hard samples using direct ink writing (DIW) is shown in Fig. 6c. To evaluate the resin/GelMA composite properties for 3D printing applications, the viscosity and shear stress of the uncrosslinked GelMA, resin, and their composites were studied using a rheometer (Fig. 6d, e). At low strain rates, the composites presented higher viscosities but lower shear stresses than at high strain rates, while the trends for different mixture ratios differ. Two composite samples of MR = 50% and MR = 75% exhibited different behaviors compared to the other samples. The MR = 50% composite presented the highest viscosity at low strain rates, followed by MR = 75%, which can be due to hydrophobic/hydrophilic interactions^74^ as also observed in SEM images (Fig. 4) where MR = 50% showed higher microsphere content, indicating stronger hydrophobic/hydrophilic interactions than other tested mixture ratios. Following the demonstration of 3D printing of this composite material, future studies will explore multimaterial direct ink writing^75^ to realize spatial tuning of stiffness and geometry. The precision and consistency of the spatial mixture ratio and stiffness in DIW could also be enhanced using active mixing nozzles^76^ in future studies.

## Conclusion

The creation of bioinspired functionally gradient structures using a hybrid diacrylate-based resin/GelMA composite has been demonstrated, where an average elastic modulus of 15 kPa on the soft side and 1.4 GPa on the hard side was obtained. This represents a change in stiffness of six orders of magnitude within the same composite material. By reducing the GelMA concentration below 10% w/v (an elastic modulus below 10 kPa), the resin/GelMA composite can even achieve a variation of seven orders of magnitude in elastic modulus, from bone-like to brain-like stiffness. This extremely wide range provides a unique platform for diverse applications in tissue phantoms and interfaces between biological tissue and medical implants. The potential for biomedical applications is also supported by the results of the cytocompatibility studies, where resin/GelMA composite has proven to be a biocompatible material. Benchmarking to other literature, Kuang et al.^14^ achieved minimum 1.4 MPa and maximum 1.2 GPa of elastic modulus using a grayscale printing technique, while Yue et al.^15^ achieved minimum 16 kPa and maximum 478 MPa of stiffness using a different resin. Using multimaterial inkjet printing and combination of liquid voxels in the structure, low modulus of 20 kPa and high modulus of 2 GPa was achieved in ref. ^69^. However, the minimum stiffness of the solid phase in that composite is more than 300 kPa. Using a composite of silicone rubber and epoxy resin, Joseph et al.^77^ achieved a broad range of 20 kPa to 2 GPa, however, biocompatibility and gradient manufacturing protocol have yet to be studied.

The direct design of complex variable stiffness structures using resin/GelMA composite is achieved by defining the spatial stiffness according to the mixture ratio. The manufacturability of these linear continuous functionally gradient materials and their demonstrated 3D printability provide a flexible pathway for the creation of arbitrary complex composite gradient interfaces for diverse in vitro and in vivo applications. These gradient stiffness interfaces can potentially be used to attach hard components to soft tissues, such as in brain-computer interfaces, or to enable integration of future implantable soft robotic technologies at the junctions between bone or cartilage and soft tissue. Future applications may also include advanced aquatic and submerged robotics, where structural stiffness tuning can enhance functionality and simplify control through morphological programming.

## Methods

### Resin

The considered hybrid resin (Water–Wash Resin+ Clear) was purchased directly from the supplier (Anycubic, China). Information about the chemical composition of resin was obtained from its safety datasheet^78^. The resin components and their volumetric ratios are: Oxirane, 2-methyl-, polymer with oxirane, bis(2-methyl-2-propenoate), block at 30–40%, Propylene glycol diacrylate at 10–25%, Tri(propylene glycol) diacrylate at 15–25%, Bisphenol A ethoxylate diacrylate at 10–20%, and photoinitiator BAPO at 2–5% of the resin’s unit volume.

#### Preparation of GelMA

GelMA was synthesized as previously reported with small modifications^46^. Briefly, 10% (w/v) homogeneous gelatin solution was prepared by dissolving the gelatin powder derived from lime-cured tissue (from bovine skin type B, G9391, Bloom  ≈225; Sigma-Aldrich) in deionized water (DW) under gentle magnetic stirring at 50 °C. Next, 0.6 mL of methacrylic anhydride (MA, ≥94%, 276685, 200 ppm topanol A; Sigma-Aldrich) per gram of dissolved gelatin was gradually mixed under continuous magnetic stirring at 50 °C for 1 h, and the mixture was then centrifuged at 3500 rpm for 3 min to remove the unreacted chemicals. The mixture was further purified by dialyzing in warm DW for 7 days using 12–14 kDa tubular dialysis membrane (14000 MWCO, 12787466, BioDesign Dialysis Tubing^TM^ (D002); Fisher Scientific) with regular change of DW. After dialysis, the pH of the mixture solution was adjusted to 7.4 using 1M sodium hydrogen carbonate solution (NaHCO3; Fisher Scientific) and then snap frozen using liquid nitrogen and lyophilized (LABCONCO, USA) for 1 week in dark conditions (covering with aluminum foil). The obtained freeze-dried GelMA prepolymer was stored at −20 °C for future use.

#### ^1^H-NMR analysis

The degree of functionalization (DOF) of gelatin was evaluated using ^1^H-Nuclear Magnetic Resonance (^1^H-NMR; Jeol-400 ECZ, Japan)^46^. For ^1^H-NMR, solutions of 10, 15, 20, and 30 mg/mL of gelatin, laboratory-synthesized GelMA, and commercially purchased GelMA were prepared in 800 μL of deuterium oxide (D2O; 99.9%, 151882, Sigma-Aldrich) at room temperature and filled in NMR tube (Norell^®^, 5 mm, NOR507HP7). The DOF in terms of methacrylation of GelMA is the percentage of free amino groups (-NH2) of gelatin modified with methacrylic anhydride, which was calculated by the peak area ratio of modified amino groups to the primary amino groups using the formula presented in Eq. 1^79^.

1 $$DOF( \% )=1-{A}_{GelMA\,lysine\,methylene}/{A}_{Gelatin\,lysine\,methylene}\times 100$$

#### FTIR analysis

Chemical structures of gelatin and GelMA were characterized using attenuated total reflectance-Fourier transform infrared spectroscopy (ATR-FTIR; System 2000, PerkinElmer, UK) over the range of 4000–500 cm^−1^ with a resolution of 4 cm^−1^ to evaluate the synthesized GelMA. The region of interest of ATR-FTIR spectra was 2000–600 cm^−1^, which presents changes in specific chemical groups. The resin/GelMA composite samples were evaluated over the range of 4000–600 cm^−1^ with a resolution of 4 cm^−1^ where the results were averaged over four repetitions.

#### MALDI-TOF mass spectrometry

MALDI-TOF mass analyses were performed on an ultrafleXtreme 2 time-of-flight mass spectrometer (Bruker Daltonics Ltd., Coventry, UK). For the composite samples, the matrix was Sinapinic acid (SA). The matrix solution was prepared by adding SA (10 mg mL^−1^ in MeCN/water 50% v/v) in a 1:1 ratio to NaCl (1 mg mL^−1^ in water with 0.1% v/v formic acid). The composite samples were then added in a 1:1 (v:v) ratio to this matrix solution, and 0.75 μL was spotted onto the target plate and dried. The M Z^−1^ range scanned was up to 50,000 but no data above 10,000 M Z^−1^ were observed in any of the samples.

#### Raman analysis

Raman spectroscopy was performed on a Renishaw 1000 system with 1200 lines/mm grating. Using the green laser at 514 nm, cured resin samples were analyzed under full excitation emission power, while liquid resin was evaluated under 33% power to prevent saturation. In all tests, spectra were acquired for 10 seconds and 20 accumulations were considered. In post-processing, baselines were corrected using Origin 2022 software.

#### Tensile test specimens

Test specimens were manufactured by injecting the liquid into the mold, sealing, and curing on the light-emitting LCD screen of a DLP 3D printer (Anycubic Photon Mono X, China) using its exposure mode. The molds were made from 2 mm thick transparent acrylic sheets, where the geometry of the specimen, the liquid inlet and outlet, and the clamping bolt holes were laser cut. The molds consist of two acrylic cover sheets that hold the sheet with the liquid inlet and specimen geometry in between. To facilitate the specimen separation during unmolding and avoid cure inhibition at the interface of the acrylic sheet and the diacrylate-based resin, FEP (Fluorinated Ethylene Propylene) films are placed between each cover sheet and mold sheet. The geometry of the specimens is according to Type 2 from ISO37 standard, where the cross-section at the thin section of the sample is 2 mm (thickness) by 4 mm (width).

The liquid was mixed and maintained at temperatures above 40 °C to avoid physical crosslinking of the GelMA component. For each mixture ratio of the composite, appropriate proportions of GelMA and resin were added to a mixing tube using a pipette. All containers used in the process, including mixing tubes, were UV-protected or fully covered by aluminum foil to avoid exposure to light and degradation before the main curing stage. Then, mixing was performed using a centrifugal mixer (ARE250, Thinky) at 2000 rpm for 2 minutes, followed by one minute of defoaming at the same speed. Immediately after mixing, the liquid was placed into a syringe and transferred into the molds. The geometry of the molds helps in avoiding air entrapment and bubbles in the specimen section.

Curing was performed using the LCD screen of the 3D printer. The light emits at 405 nm wavelength, and the total time for curing the samples was 500 s, i.e., 250 seconds for each side. The emitted light power was measured using a light meter (Chitu Systems, China), indicating 7.65 mW cm^−2^ on the LCD Surface. However, the net light power reaching the material is reduced due to its passage through the 2 mm thick acrylic sheet and FEP film of the mold, resulting in 5.73 mW cm^−2^ of effective light power.

The pure resin test specimens (ISO37 Type 2) were 3D printed instead of molding because the pure resin has an exothermic reaction during curing, which generates considerable heat, resulting in wrinkles in the FEP film and surface waviness or cracking in the sample. For 3D printing, the light power at the surface passing through one layer of FEP film (no acrylic mold was used for the 3D-printed specimens) is 7.4 mW cm^−2^ and the curing time for each layer is 3 seconds and each layer is 50 micron in height. After printing, samples are washed with isopropanol and post-cured for 10 minutes (5 minutes each side) in the cure station (Wash and cure station plus, Anycubic, China). The light power measured at the surface of the specimen in the post-curing stage was 3.9 mW cm^−2^.

#### Tensile test

Tensile tests were performed using a universal tensile testing machine (AGS-X series, Shimadzu, Japan) with two sets of load cells. A 5 N load cell (Shimadzu, Japan) was used for testing GelMA and 20% composite samples, and a 500 N load cell (Shimadzu, Japan) was used for all other composite and resin samples. All tests were performed at 2 mm s^−1^ speed and samples were tested immediately after unmolding to avoid moisture content reduction. To avoid slippage of the samples in the grippers of the test machine, a piece of sandpaper was installed between the gripper jaws. Strain was measured using a video extensometer (Imetrum, UK), including a 5 MP camera with 25 mm focal length.

#### FE-SEM analysis

Field emission-scanning electron microscopy (FE-SEM) for resin/GelMA composites (10%, 50%, and 75%) and pure resin samples was performed at the Wolfson BioImaging Facility at the University of Bristol on an FEI Quanta 200 FE-SEM. For FE-SEM of pure resin and composite samples, a freeze-dried sample was platinum-coated (Emitech K550X Sputter, USA) before imaging in the SEM.

#### Cell culture samples

The cell culture samples were manufactured in a similar way as tensile test specimens (GelMA and composites molded and pure resin samples 3D printed), while the mold geometry was modified to produce 2 mm thick circles of 8 mm diameter. It is important to wash the samples using ethanol and PBS several times and place them in PBS for several days to remove the unreacted chemicals from the hybrid resin and the photoinitiator.

The cytotoxic effects of unreacted BAPO were minimized through an empirical approach. Fabricated resin/GelMA composite samples were repeatedly tested for cellular activity to determine the optimal washing duration for removing residual photoinitiator. Initially, samples were washed with PBS for 24 hours, then extended to 3, 5, and 7 days. While the 24-hour and 3-day washed samples exhibited low cell viability, those washed for 5 and 7 days showed significantly improved viability. For these tests, samples (diameter = 8 mm) were immersed in 1 mL of PBS, incubated at 37 °C, and the PBS was refreshed every 24 hours.

The cellular activities of resin/GelMA composite samples were evaluated in terms of cell viability, cell proliferation, and metabolic activity using LIVE/DEAD assay, DAPI/Phalloidin staining, and AlamarBlue assay, respectively, for up to 5 days. The cell culture work was carried out in a SAFE 2020 laminar flow hood (Thermo Fisher Scientific, UK). Cells were incubated in a Culture Safe Precision P190D incubator (LEEC, UK) at 37 °C, 5% CO2 atmosphere. C3H mouse myoblast cell line C2C12 was purchased from Merck, UK, and cultured in Dulbecco’s Modified Eagle’s Medium high-glucose medium (Merck, UK) with 10% (v/v) of fetal bovine serum (FBS), and 1% (v/v) of penicillin/streptomycin as a supplement. Cells were passaged once they reached ~70–80% confluency using trypsin/EDA solution (Sigma-Aldrich, UK) as recommended by the manufacturer's guidelines.

Prior to cell seeding on resin/GelMA composites, samples were thoroughly washed trice with ethanol (70%, and 80%) and phosphate buffer saline (PBS). Finally, samples were kept in PBS solution for 5 days at 4 °C, and the solution was changed every day to clear unreacted resin components and photoinitiator. C2C12 cells (passage 4) of 1 × 10^4^ cells per sample were seeded in a 48-well plate and cultured for 1, 3, and 5 days. The cell viability was analyzed by LIVE/DEAD^TM^ cell imaging kit (Thermo Fisher Scientific; Invitrogen, L3224, UK) at day 1, day 3, and Day 5. The cell imaging was performed on a confocal laser scanning microscope (Leica SP8 AOBS) attached to a Leica DMi8 inverted epifluorescence microscope (Leica, UK) at the Wolfson Bioimaging Facility within the Faculty of Life Sciences at the University of Bristol, UK. The images were captured using Leica LAS-X acquisition software (Leica, UK) and were processed using Fiji software.

Cell attachment, spreading morphology, and cell proliferation were assessed by staining cell nuclei (DAPI) and cytoskeleton (Phalloidin). After cell culturing on resin/GelMA samples for the defined time points (1, 3, and 5 days), cells were fixed in 4% paraformaldehyde (PFA; Fisher Scientific; 15670799, UK) solution for 10 min and washed trice with PBS. This was followed by cell permeabilization using 0.1% Triton X-100 (Sigma-Aldrich; T8787) for 10 min, washing three times with PBS, and blocking with 2% of bovine serum albumin (BSA; A3294, Sigma-Aldrich) for 1 h at room temperature. Further, the cytoskeleton and nuclei were stained with phalloidin (Thermo Fisher Scientific; Invitrogen, Alexa Fluor™ 546, A22283, USA), and (DAPI; Thermo Fisher Scientific; Invitrogen, 10184322, USA), respectively.

Metabolic activity was measured using AlamarBlue (Thermo Fisher Scientific; Invitrogen, DAL1025, UK). The AlamarBlue dye was diluted with serum-free cell culture media and added 10% v/v to each sample, and incubated for 3 h at 37 °C in the dark. The reacted supernatant was transferred to a 96-well plate (Corning^®^; Labwares, UK) and the fluorescent intensity of each well was measured using a fluorescence multiplate reader at excitation/emission wavelength 530/590 nm. The fluorescence intensity was normalized using the reduced AlamarBlue compared with the tissue culture plate (TCP) control. This allowed calculation of relative cell metabolic activity.

#### Gradient manufacturing

The gradient sample was manufactured by injecting the liquid into a rectangular mold. The mold was made from a transparent acrylic sheet, and FEP film was placed between the cover sheet and the mold, where the rectangular sample dimensions were 35 mm × 35 mm × 3 mm. Two syringe pumps (AL1000, World Precision Instruments, USA) were used synchronously to control the flow rate of the two syringes, one filled with GelMA and the other with resin. The syringes were covered by aluminum foil to avoid light exposure and curing. The mold was installed on a vertically-oriented stage connected to a linear actuator (NEMA17-LA-C, Ooznest, UK) that moved the mold down as it filled up. The linear actuator and the two syringe pumps were synchronized and controlled by an Arduino controller (uStepper, Denmark).

To mix the resin and GelMA, a static mixing tip (Universal mixing tip, Coltene, Switzerland) was used. The static mixing tip generates a resistance to inflow, which could cause injection of the resin into the GelMA syringe instead of the mixing tip. To mitigate this, a flow check valve was used. Custom-built adapters for the mixing tip inlet and outlet were 3D printed using resin (Translucent green, Anycubic, China) and a DLP printer (Photon Mono X, Anycubic, China). At the outlet, a 14G and 38 mm long blunt tip dispensing needle (Muzamedical, UK) was used, which enables the tip to reach the bottom of the mold at the beginning of the injection. The volume of the liquid in the mixing tip is about 1 ml, which affects the resolution of the gradient manufacturing.

#### Hardness measurement

The hardness was measured using two durometers: Shore-A scale (RS PRO Durometer, RS, UK) and Shore-00 scale (Sauter HB0 100-0 Durometer, Sauter, Germany). Specific frames and adapters for the durometers were designed and 3D printed to allow the solid installation of the durometers on a precision stage (Thorlabs, USA). On the harder side and using Shore-A durometer, the gradient sample’s hardness was measured at every 2 mm step. On the softer side and using Shore-00 durometer, hardness was measured at every 3 mm distance because of the larger diameter of the Shore-00 indenter. The hardness of the pure resin and pure GelMA were measured separately, where the resin and GelMA were molded and photocured. The mold was a cylinder with 19 mm diameter and 6 mm height.

#### Rheology

Dynamic viscosity was measured using a rheometer (MCR 302, Anton Paar, Austria) with a 25 mm (diameter) aluminum parallel plate with a gap distance of 1 mm. Rotational tests were conducted with strain rates varying between 0.01–1000 s^−1^. All samples were tested at 30 °C in their liquid states. The viscosity was given by the following equation $\mu =\tau /\dot{\gamma }$, where *μ*, *τ*, and $\dot{\gamma }$ are viscosity, shear stress and shear strain rate, respectively. Resin, GelMA, and their composites show no sudden change in stress values or slope of stress-strain rate curve over the range of test and the rheometer measured stress at the low end of the machine’s torque capacity. The stress values at low strain rate are quite small (less than 0.1 Pa), and a very low yield stress at the test temperature is expected.

#### 3D printing

DIW was performed using a BioX 3D printer (Cellink, USA) with GelMA (10% w/v, using 22 Gauge needle, 30 °C temperature, 5 kPa pressure) and resin (using 22-gauge needle, 25 °C temperature, 7 kPa pressure). To maintain shape fidelity during printing, each layer was cured separately, where curing between each layer was conducted using an onboard 405 nm LED for 3 seconds. After finishing the printing, the sample was post-cured for 500 seconds in the curing station (Wash and cure station plus, Anycubic, China) to fully crosslink the GelMA.

## Supplementary information

Supplementary Material

Description of Additional Supplementary File

Supplementary Video

## Data Availability

Data supporting this work is available for download from the University of Bristol Research Data Repository through the following link: 10.5523/1s6nyn45o8fzd2jrsonnf97qq4.
