# Supplementary material for: Hybrid diacrylate resin-gelatin methacryloyl composite with bone-to-brain stiffness range
Source: Commun Mater. 2025 Oct 2;6(1):219. doi: 10.1038/s43246-025-00931-y (PMC12542801; doi:10.1038/s43246-025-00931-y)
Supplement: Supplementary file 1 — Supplementary Material [file 43246_2025_931_MOESM1_ESM.pdf]

Hybrid diacrylate resin-gelatin methacryloyl composite with bone-to-brain stiffness range

Mohammad Naghavi Zadeh*, Kapil D. Patel*, Daniel Gosden, James A. Smith, Paul J. Gates, Qiukai Qi, Fabrizio Scarpa, Andrew Conn, Adam W. Perriman, Jonathan Rossiter

Supplementary Information

Supplementary information 1: Studying the bond line between GelMA and resin

The bond line between GelMA and resin in an open mold and after curing is shown in Supplementary Figure 1a. To evaluate the chemical bonding strength between the resin and GelMA, tensile testing was performed. For this test, a transparent closed mold that provides constant thickness over the sample with a removable barrier in between was manufactured. From one side resin and from the opposite side GelMA were injected. Then, the barrier (0.4mm thickness plate made from black PETG) was removed to allow for resin and GelMA diffusion happen at the interface for 10 seconds and then curing on the LCD surface of the printer began. Tensile tests show that failure in two samples occurred at the GelMA side close to the interface line proving the chemical bonding/networking that exists between GelMA and resin is stronger than crosslinks within GelMA.

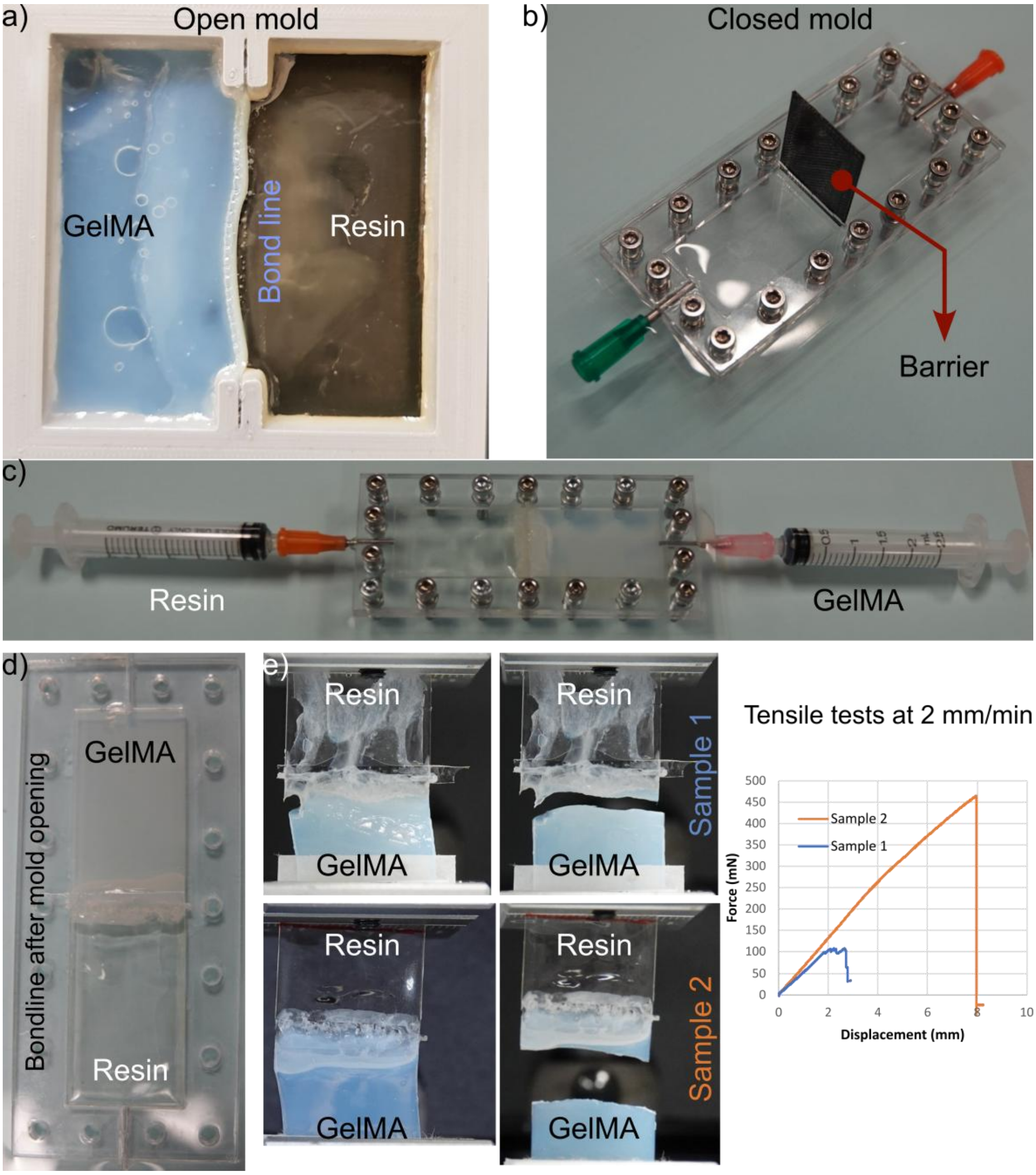

Supplementary Figure 1. (a) Resin/GelMA bond line is shown in an open mold, (b) the geometry of a closed mold showing removeable barrier, (c) resin/GelMA bond line after cure, (d) the bond line after opening the mold and diffusion area, and (e) tensile test of the samples.

Supplementary information 2: Mechanical testing details

The tensile test setup is shown in Supplementary Figure 2a including the video extensometer camera. The mold's inlet (Supplementary Figure 2b) is designed to inject the liquid from the top flowing all the way down and start filling the mold section from bottom to avoid any air trap and bubble generation during injection.

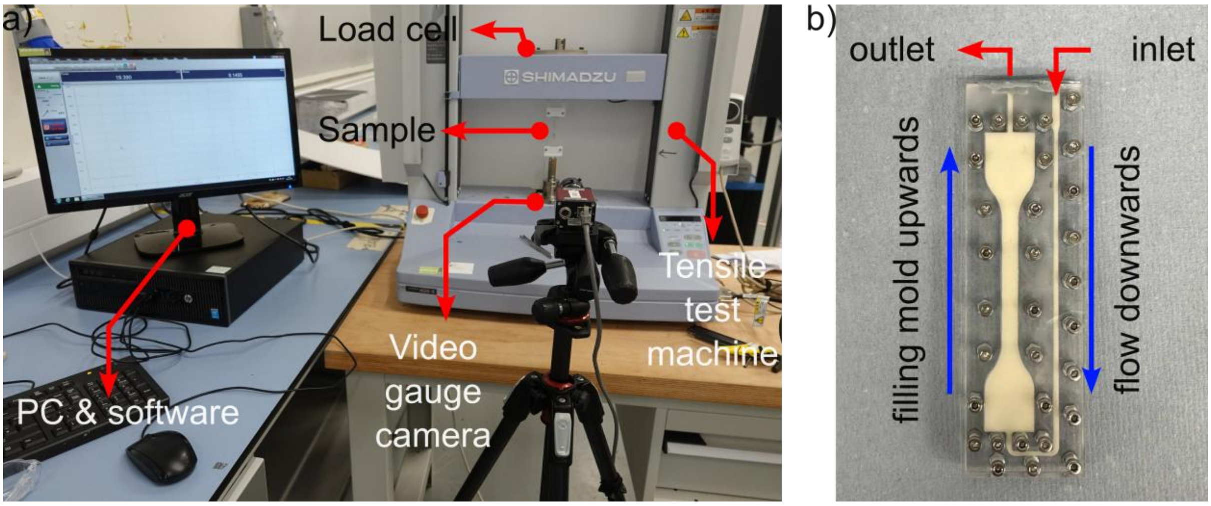

Supplementary Figure 2. (a) Tensile test setup including Shimadzu AGS-X machine, load cell, sample, Imetrum video gauge camera, and PC for control software, and (b) geometry of the mold and sample after cure but before unmolding.

The geometrical parameters and dimensions of the ISO37 Type 2 samples are shown in Supplementary Table 1.

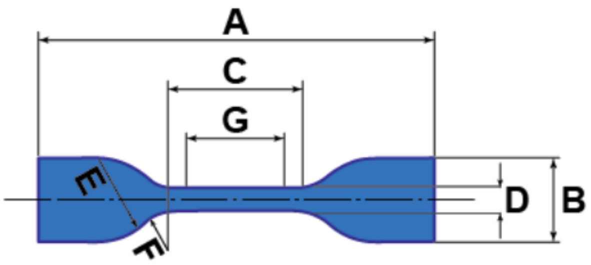

Parameter	ISO 37 Type 2 dimensions (mm)
Overall length (A)	75
Width of the end tab (B)	12.5
Length of narrow section (C)	25
Width of narrow section (D)	4
Outside transition radius (E)	8
Inside transition radius (F)	12.5
Gauge length (G)	20
Thickness (t)	2

Supplementary Table 1- Dimensions of geometrical parameters for an ISO 37 Type 2 tensile test specimen.

Samples of GelMA, resin, and composites with mixture ratio (MR) = 20%, 40%, 50%, 60%, and 80% used for hardness measurement (Supplementary Figure 3a) and durometry setup for gradient sample (Supplementary Figure 3b) are illustrated. The samples are shown after 7 days of immersion in deionized water where pure GelMA sample has expanded due to water

absorption. The samples have a circular cylindrical geometry where pure resin and pure GelMA are molded with 19 mm diameter in 6 mm thickness molds. The composite samples are molded with lower thickness of 3 mm (similar to the gradient sample) due to transparency issue and light exposure challenges. It is visually observed that increase in the mixture ratio increases the yellow color hue in the sample.

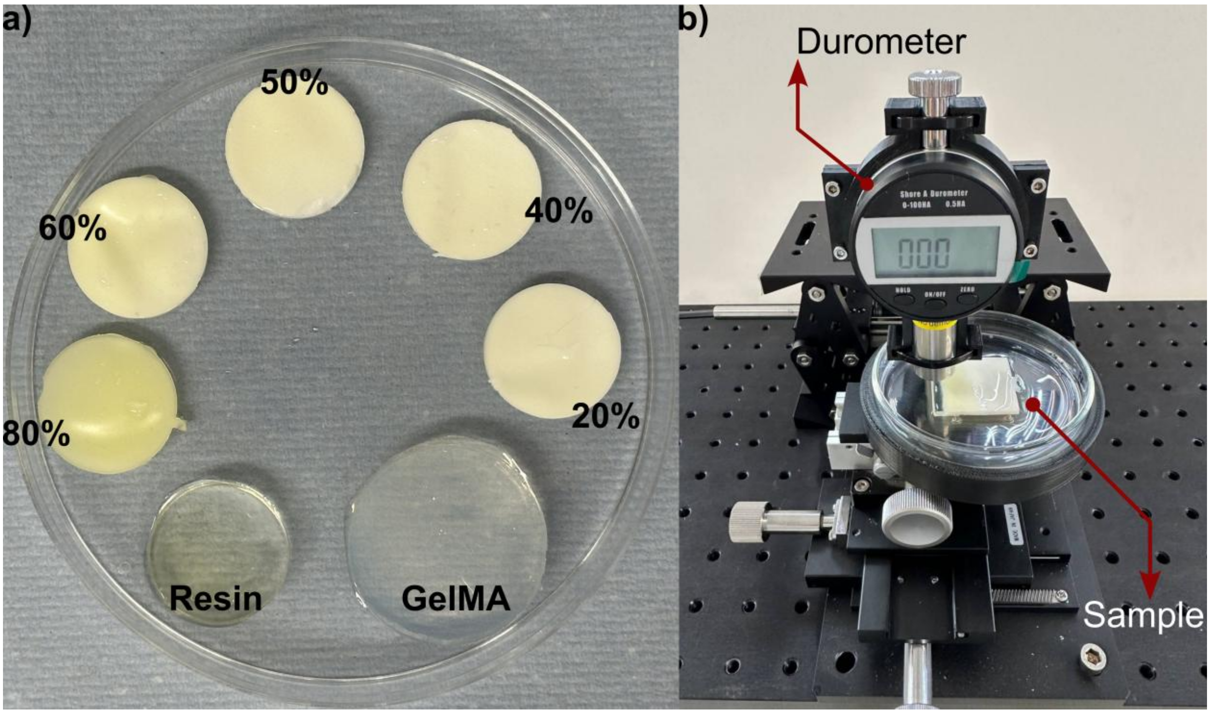

Supplementary Figure 3. (a) Cylindrical samples made for Shore hardness measurement, and (b) hardness measurement setup for the gradient sample with precision stage.

The hardness values of the samples with different mixture ratios are presented in the diagram below where hardness was measured immediately after samples were cured. Composite sample with 20% mixture ratio presents zero Shore A, therefore we switched to Shore 00 where 35 Shore 00 was obtained. The pure GelMA sample is quite soft and presents zero Shore 00. It is evident that the hardness in Shore A scale has a nonlinear but strictly monotonic relation with mixture ratio.

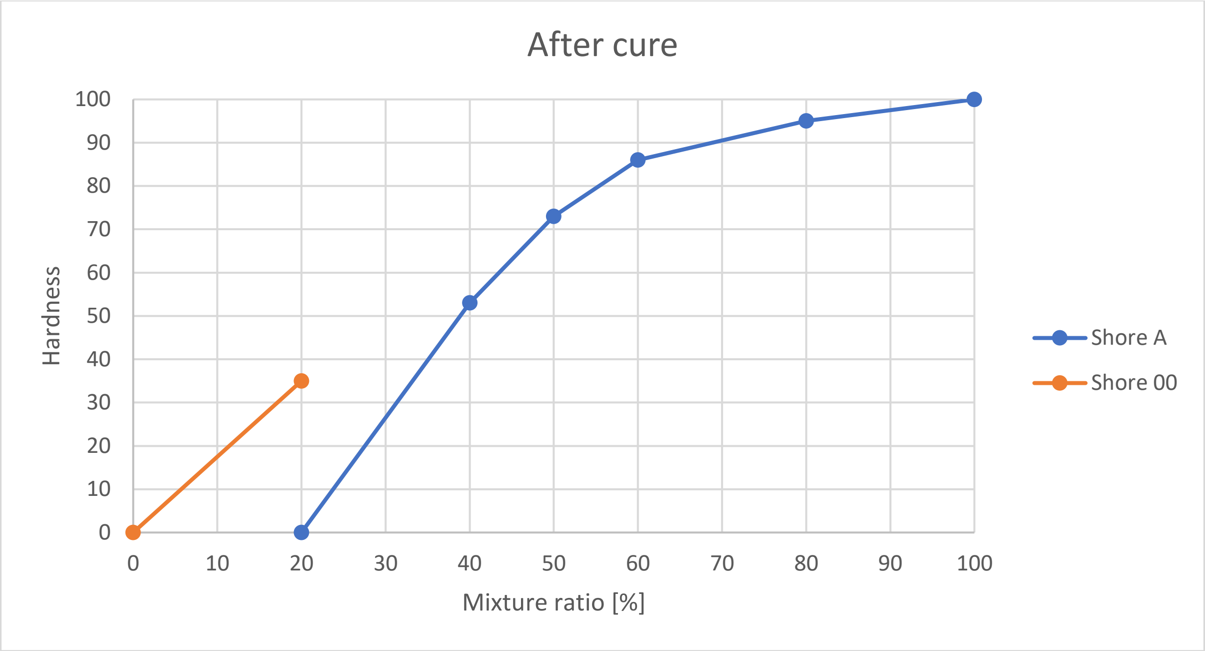

Supplementary Figure 4. Shore hardness values of resin, GelMA, and composite samples.

Supplementary information 3: Resin/GelMA Biocompatibility study details

The mold used to make the samples for biocompatibility tests is shown in Supplementary Figure 5.

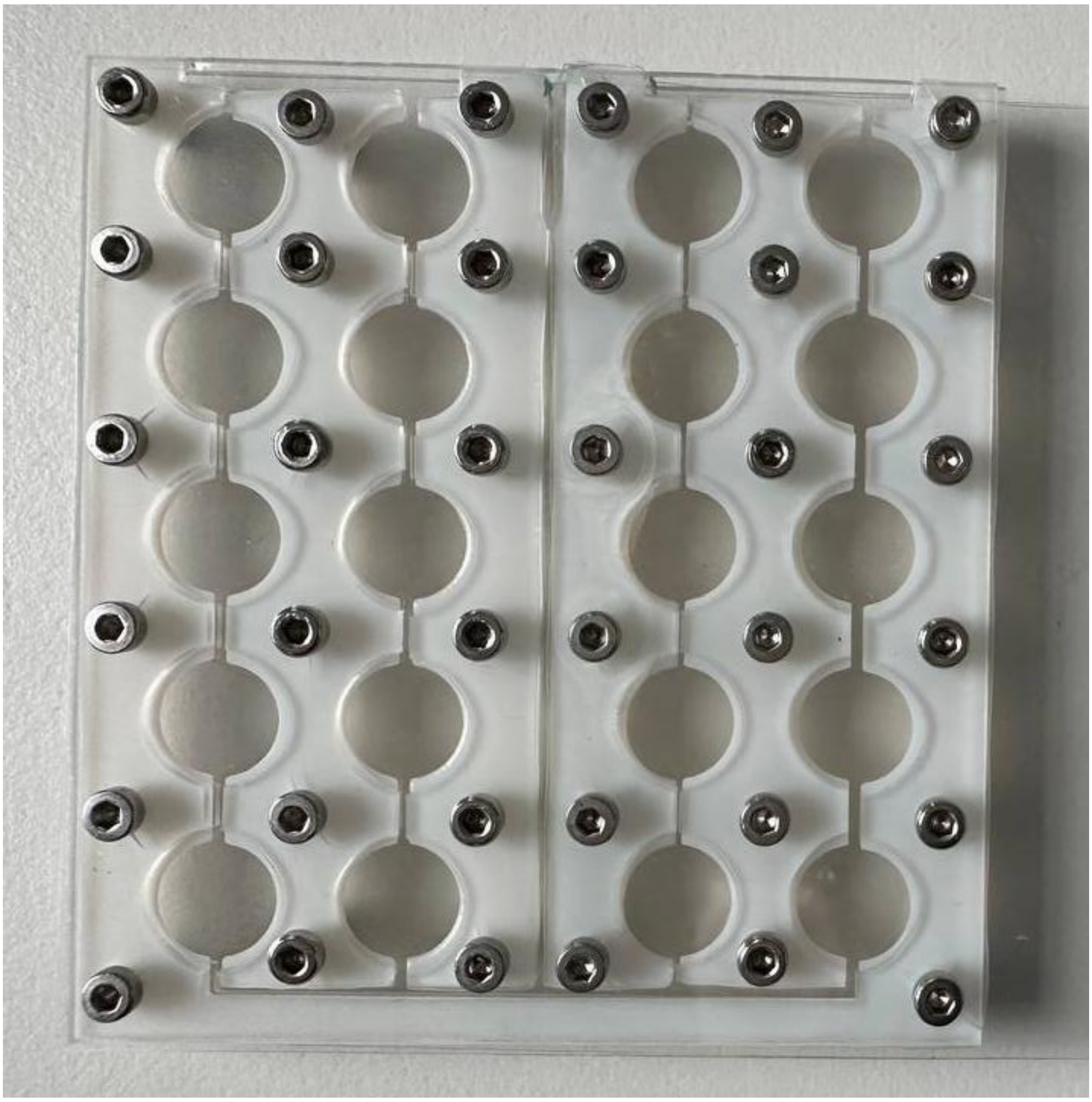

Supplementary Figure 5. The geometry of the mold used for making biocompatibility test samples.

The samples on the tissue plate are shown in Supplementary Figure 6.

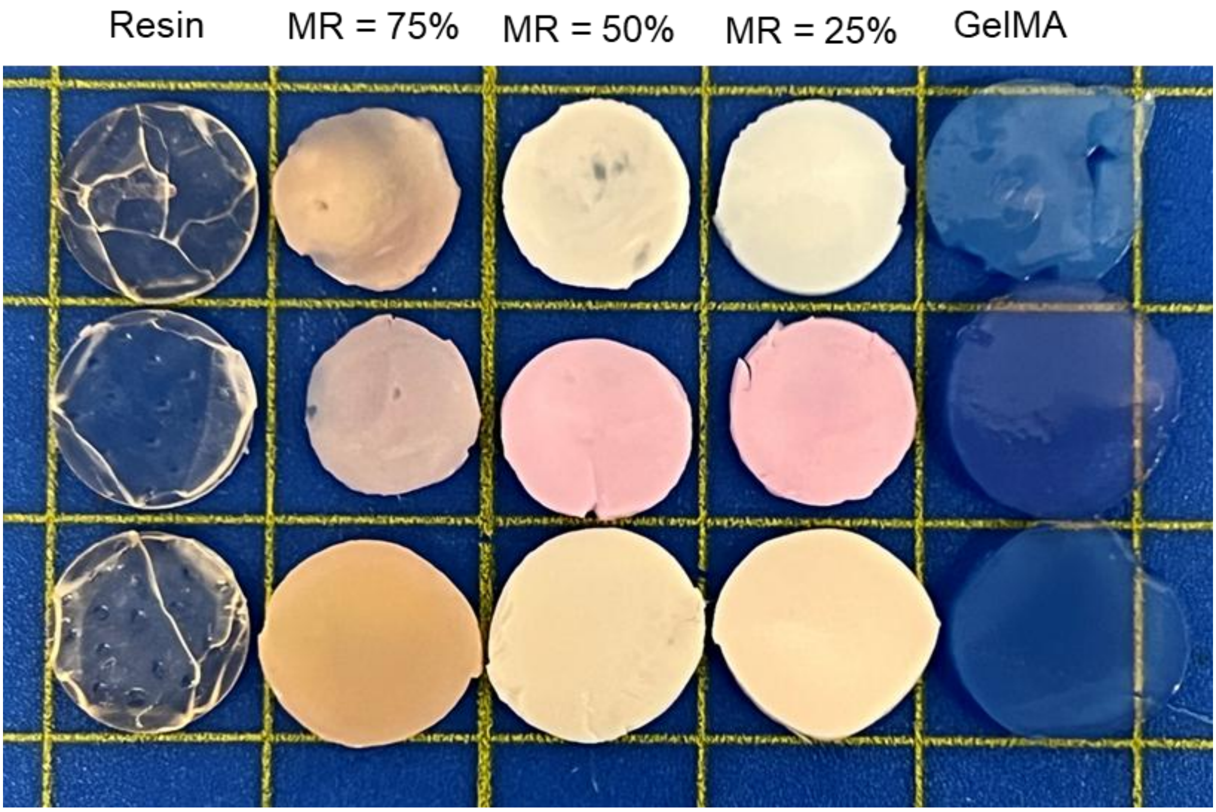

Supplementary Figure 6. One example of a tissue culture plate (TCP) with samples of different mixture ratios used for biocompatibility evaluation.

The biocompatibility study parameters were:

- Cell type: C2C12 (P#9)
- Media: DMEM high glucose (1% PS, 10% FBS)
- Cell number: 0.5 x 10^4 Cell/well
- Control: Tissue culture plate (TCP)
- Study periods: Day 1, Day 3, and Day 5
- Number of samples: Triplicated (n=6)
- Studies: Cell viability (LIVE/DEAD), Cell metabolic activity (AlamarBlue), and Proliferation (DAPI/Phalloidin)

Cell viability study

Cell viability studies were performed on day 1, day 3, and day 5 after cell culture using Live/Dead assay. The confocal microscopy images are provided for two magnifications in Supplementary Figure 7 to 12.

Day 1

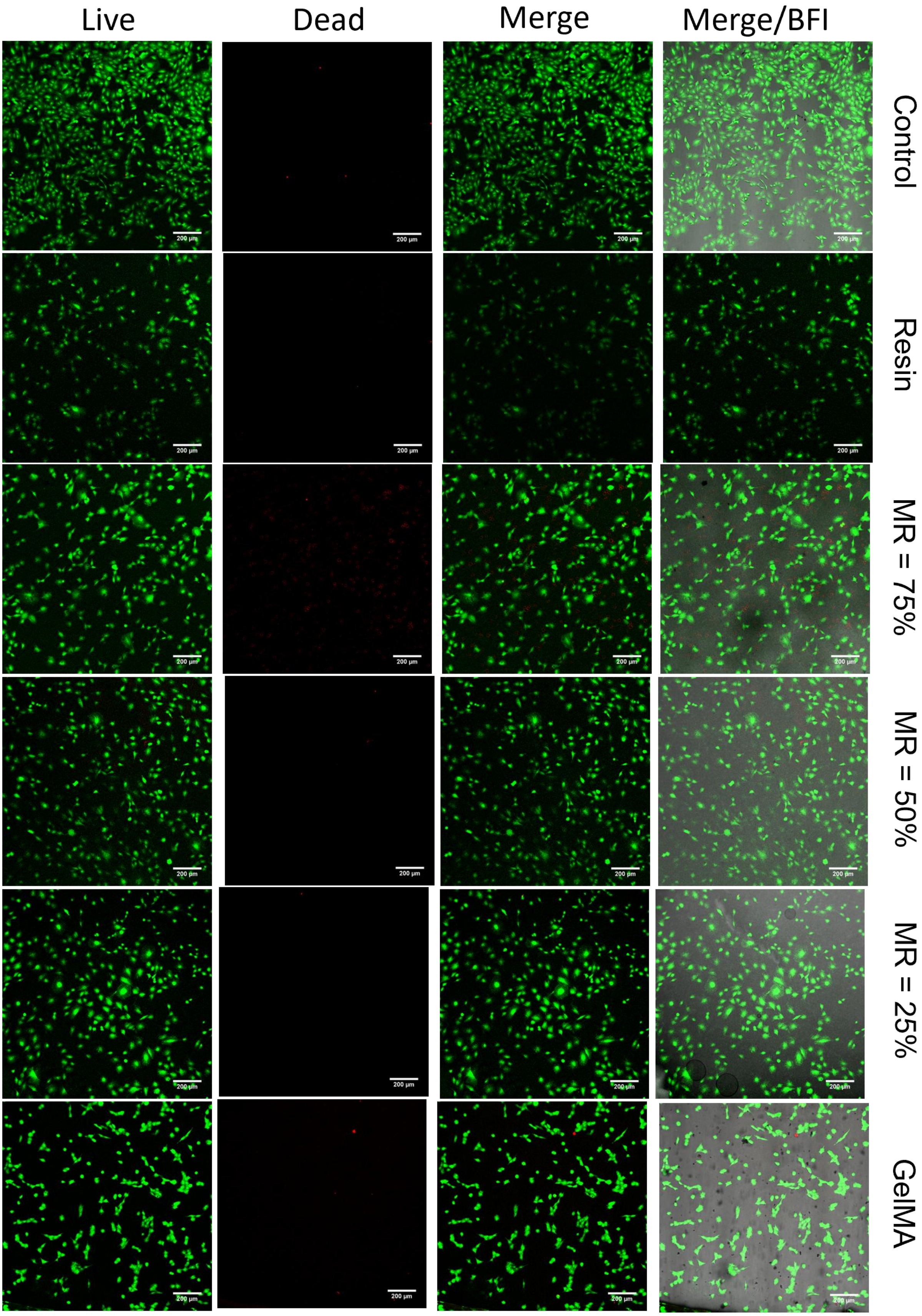

Supplementary Figure 7. Day 1 cell viability results for different mixture ratios including bright field images (BFI). Scale bar is 200 micron.

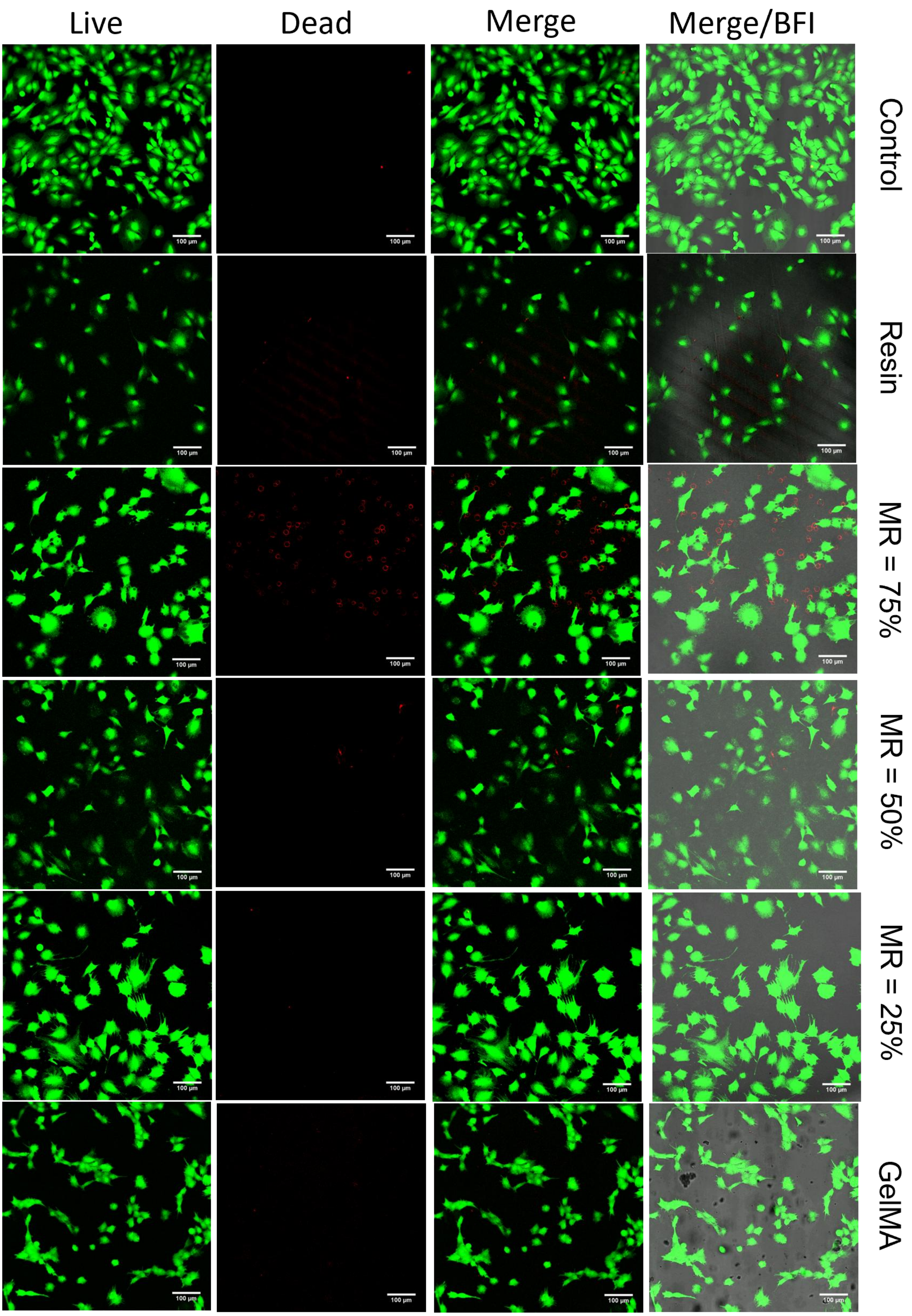

Supplementary Figure 8. Day 1 cell viability results for different mixture ratios including bright field images (BFI). Scale bar is 100 micron.

Day 3

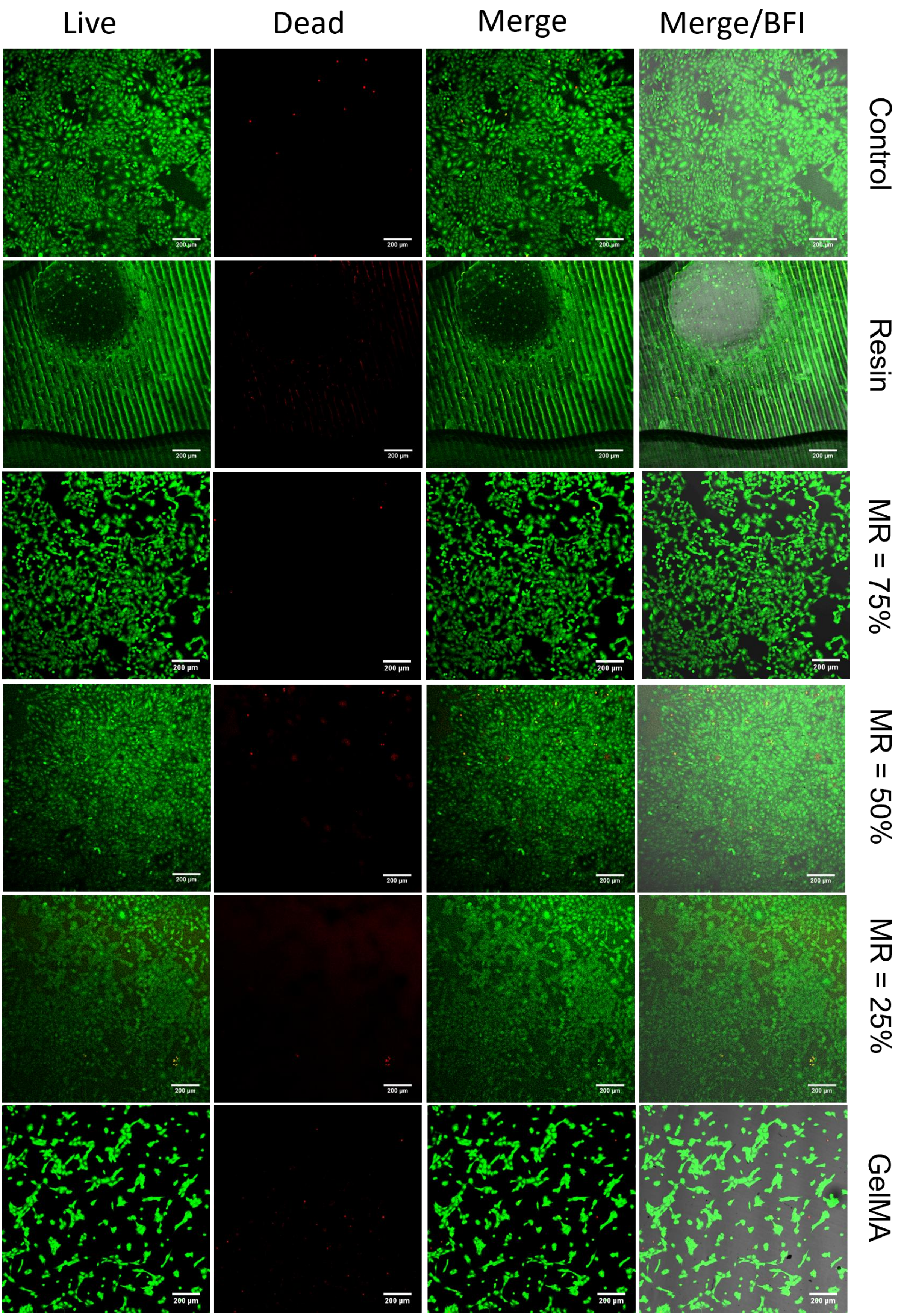

Supplementary Figure 9. Day 3 cell viability results for different mixture ratios including bright field images (BFI). Scale bar is 200 micron.

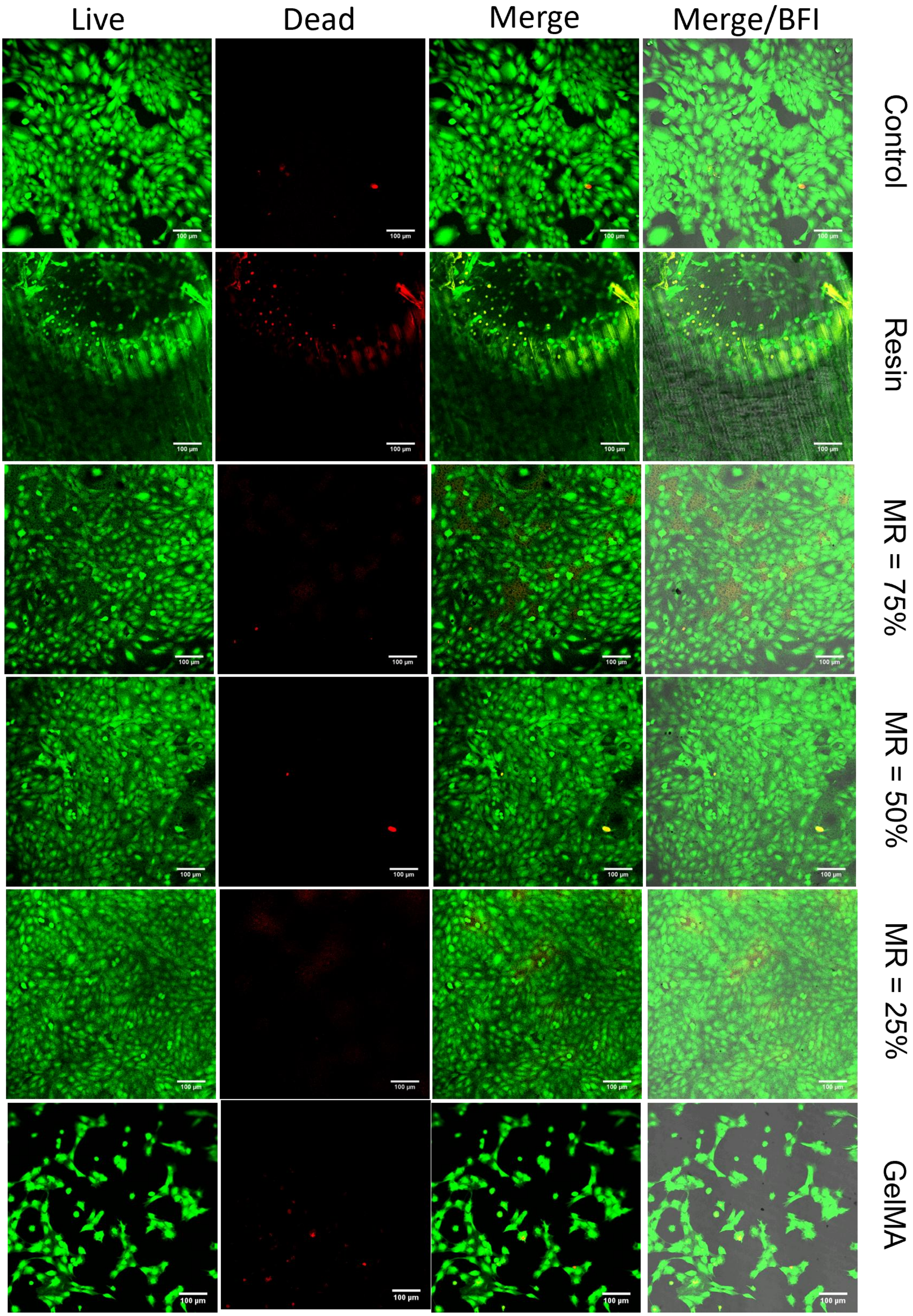

Supplementary Figure 10. Day 3 cell viability results for different mixture ratios including bright field images (BFI). Scale bar is 100 micron.

Day 5

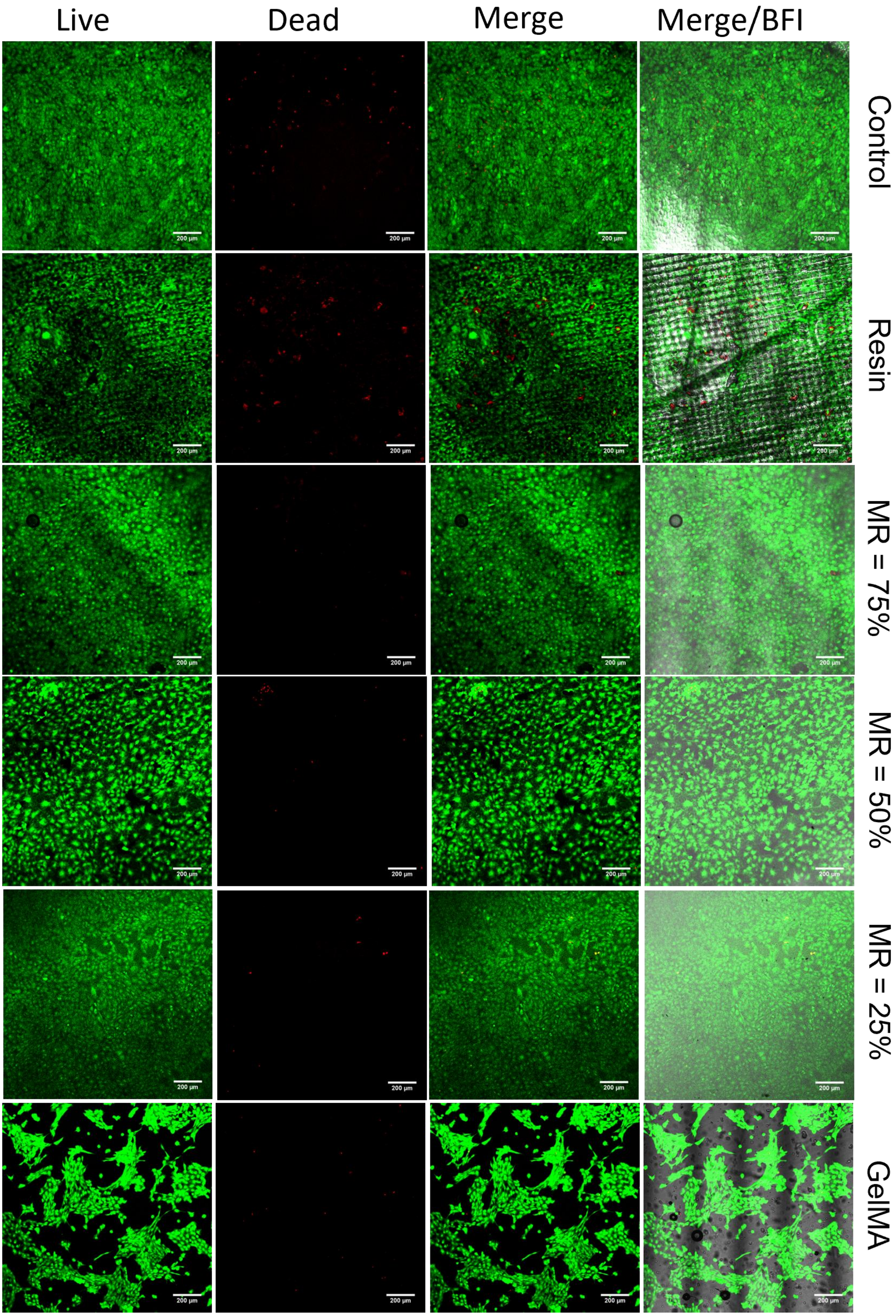

Supplementary Figure 11. Day 5 cell viability results for different mixture ratios including bright field images (BFI). Scale bar is 200 micron.

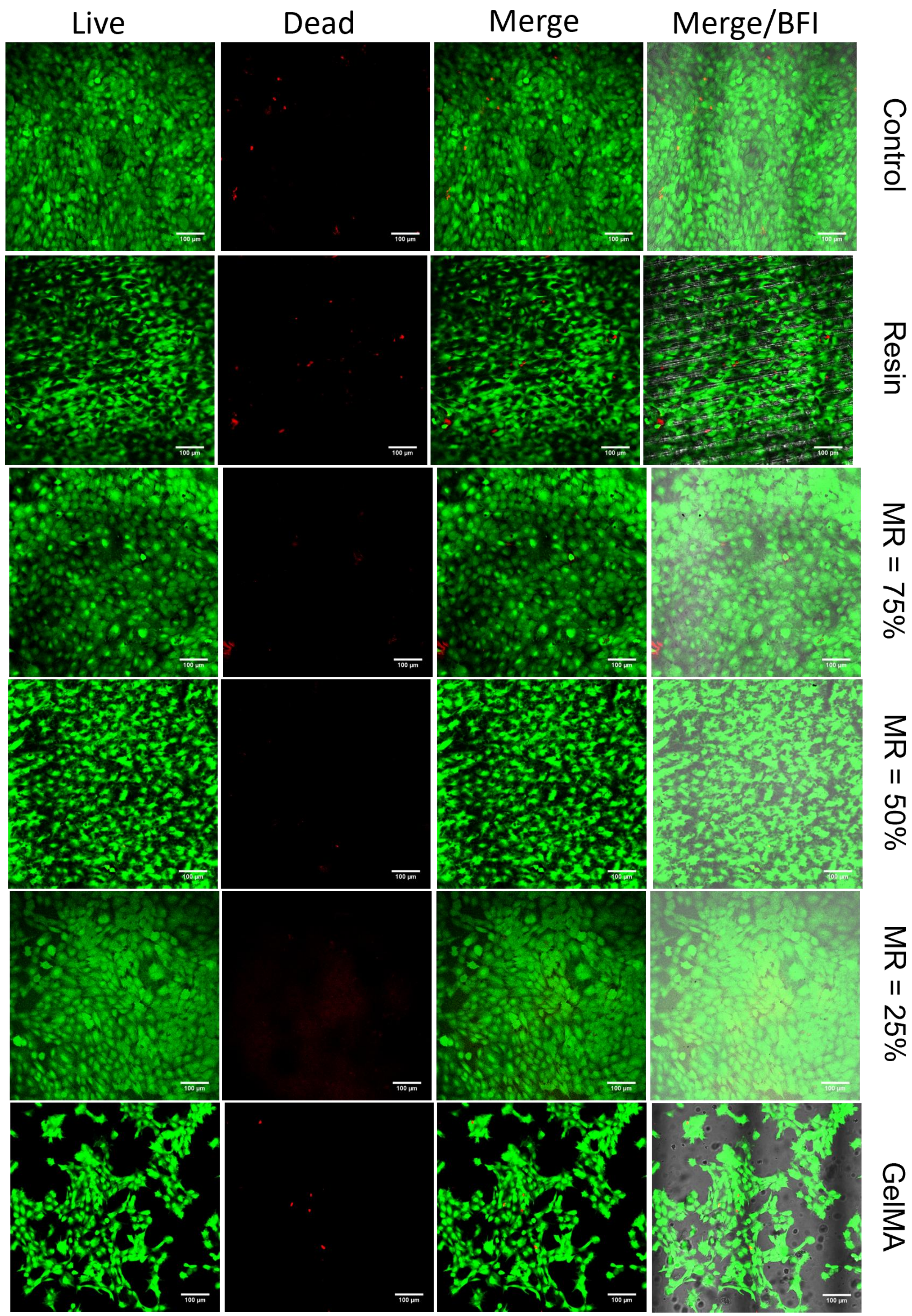

Supplementary Figure 12. Day 5 cell viability results for different mixture ratios including bright field images (BFI). Scale bar is 100 micron.

Cell proliferation study (DAPI/Phalloidin staining)

Day 1

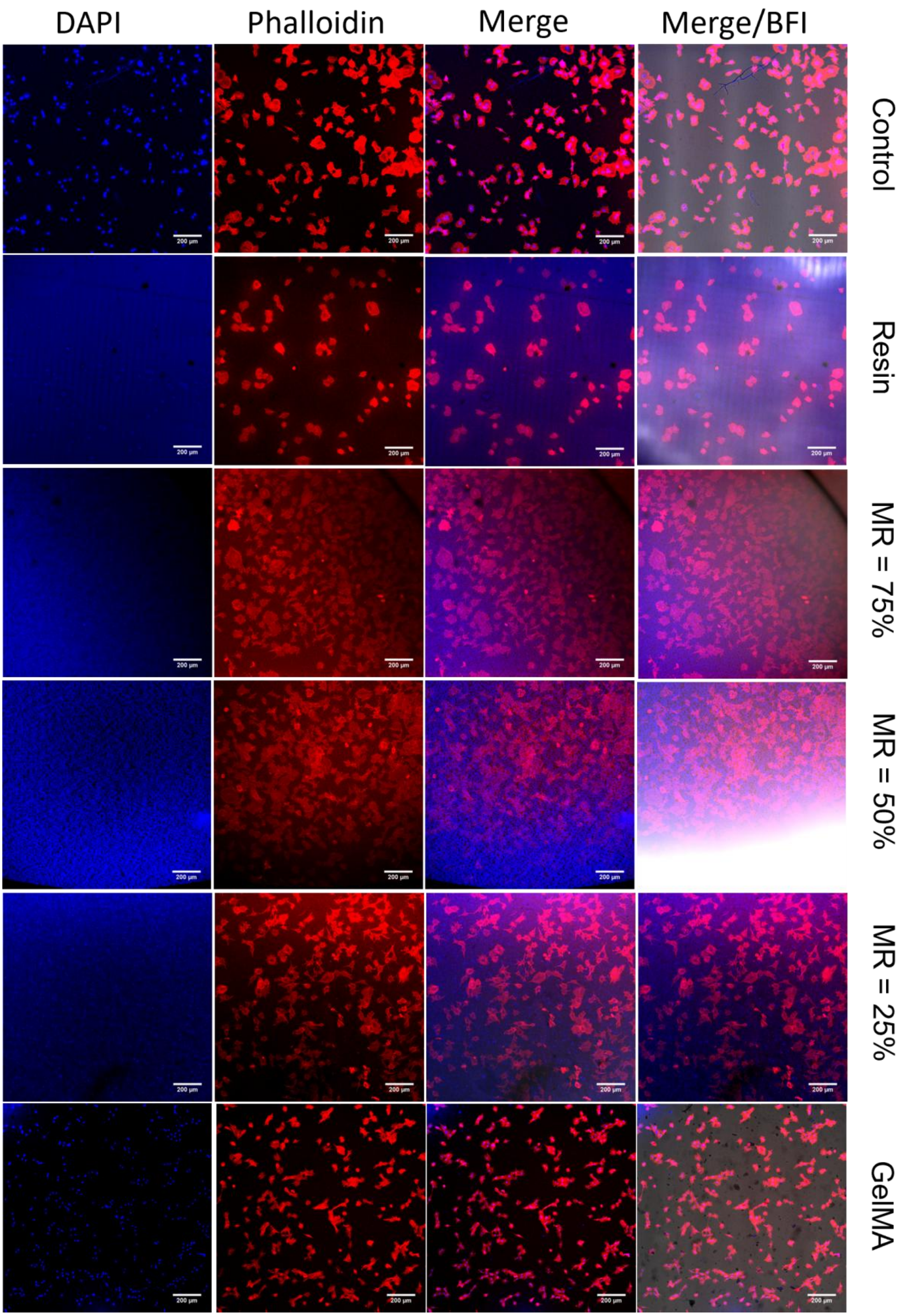

Supplementary Figure 13. Day 1 cell proliferation results for different mixture ratios including bright field images (BFI). Scale bar is 200 micron.

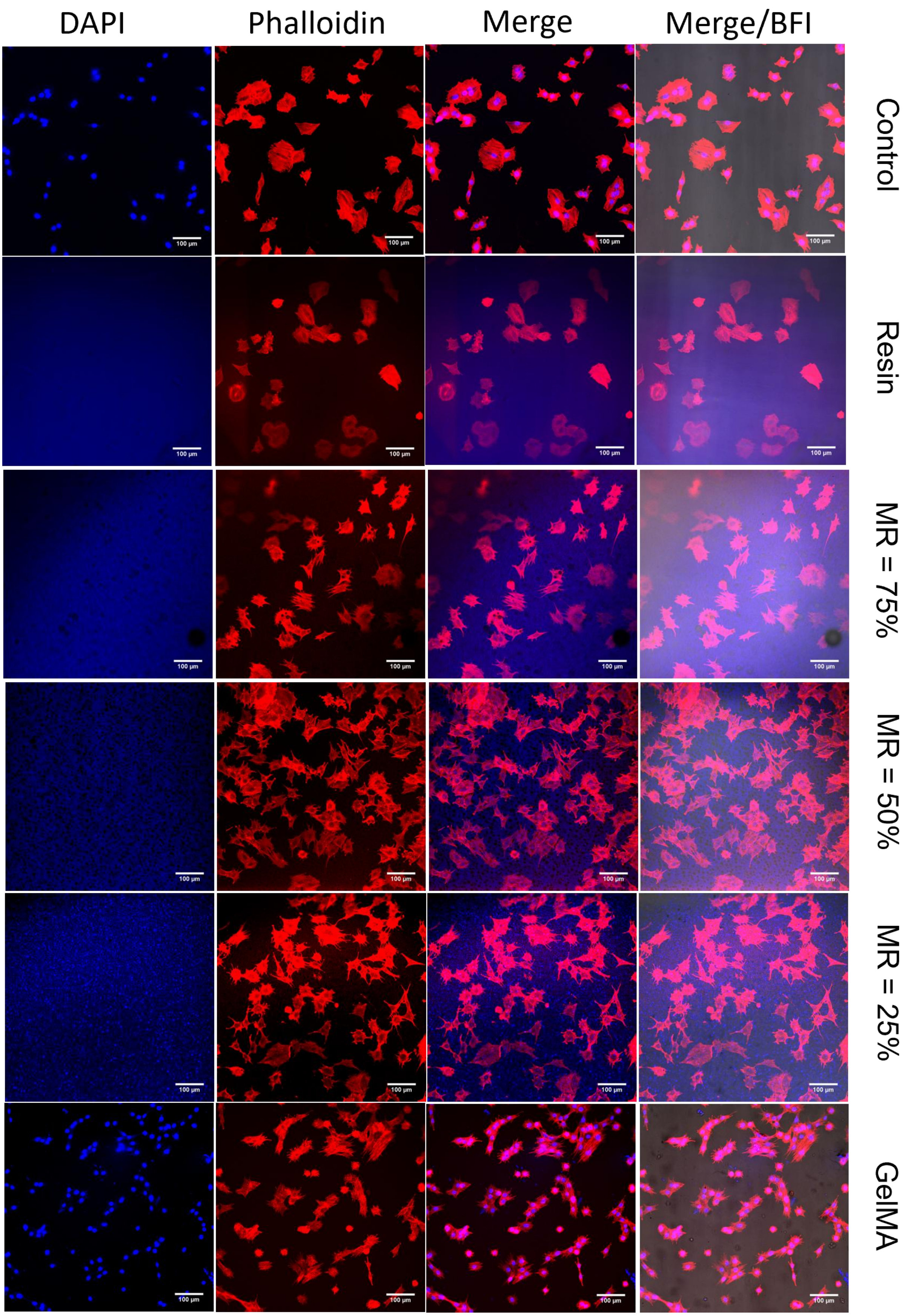

Supplementary Figure 14. Day 1 cell proliferation results for different mixture ratios including bright field images (BFI). Scale bar is 100 micron.

Day 3

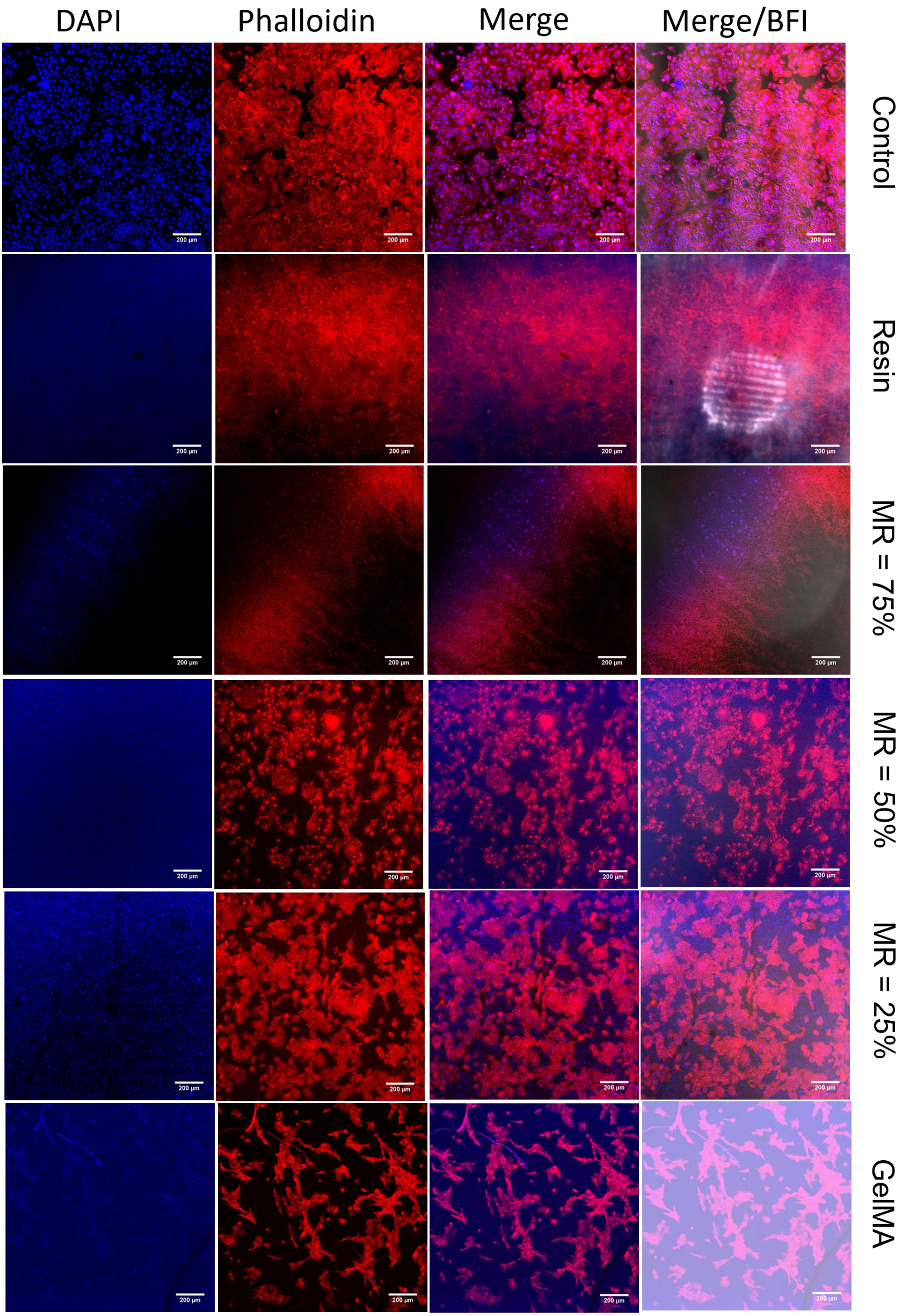

Supplementary Figure 15. Day 3 cell proliferation results for different mixture ratios including bright field images (BFI). Scale bar is 200 micron.

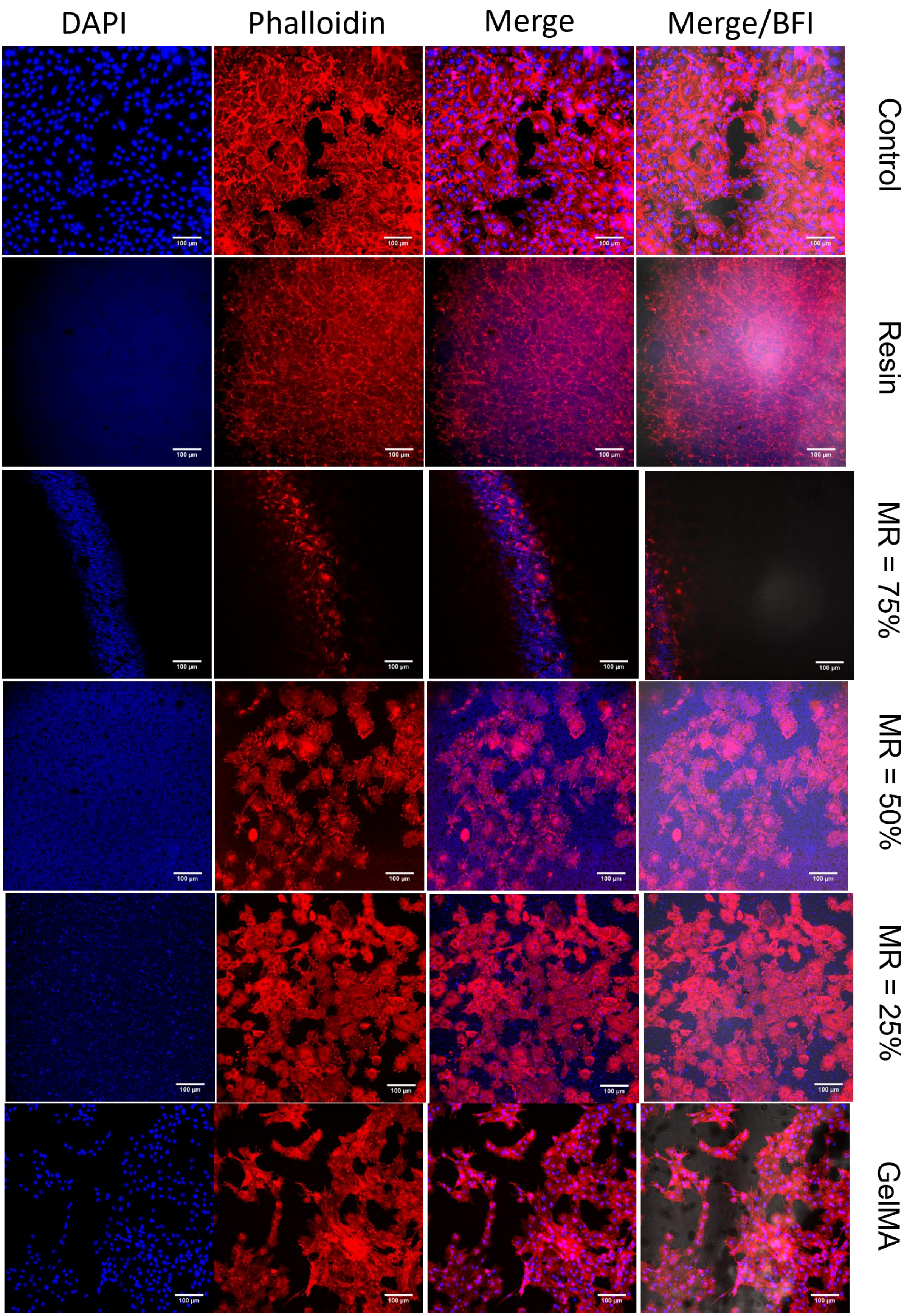

Supplementary Figure 16. Day 3 cell proliferation results for different mixture ratios including bright field images (BFI). Scale bar is 100 micron.

Day 5

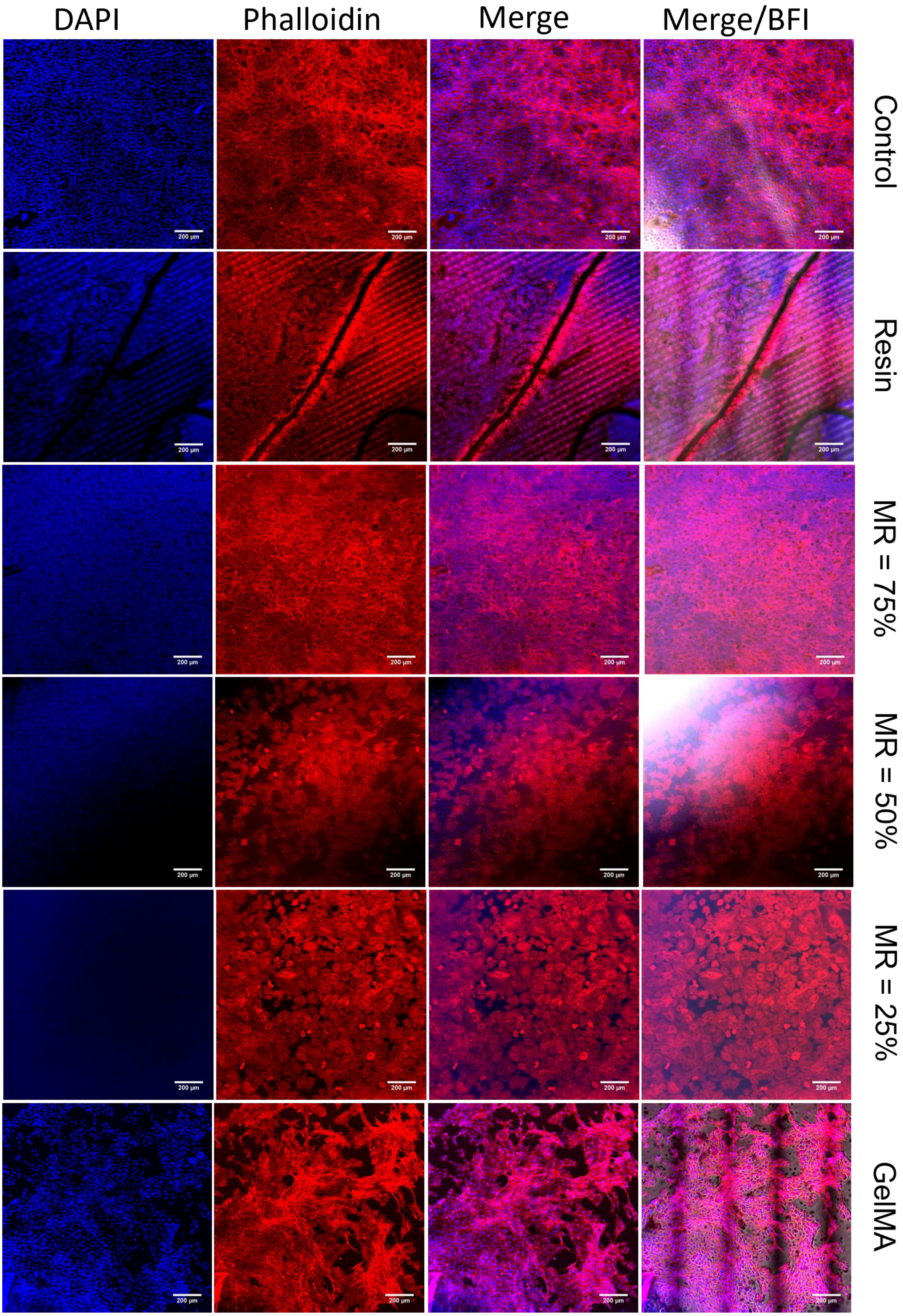

Supplementary Figure 17. Day 5 cell proliferation results for different mixture ratios including bright field images (BFI). Scale bar is 200 micron.

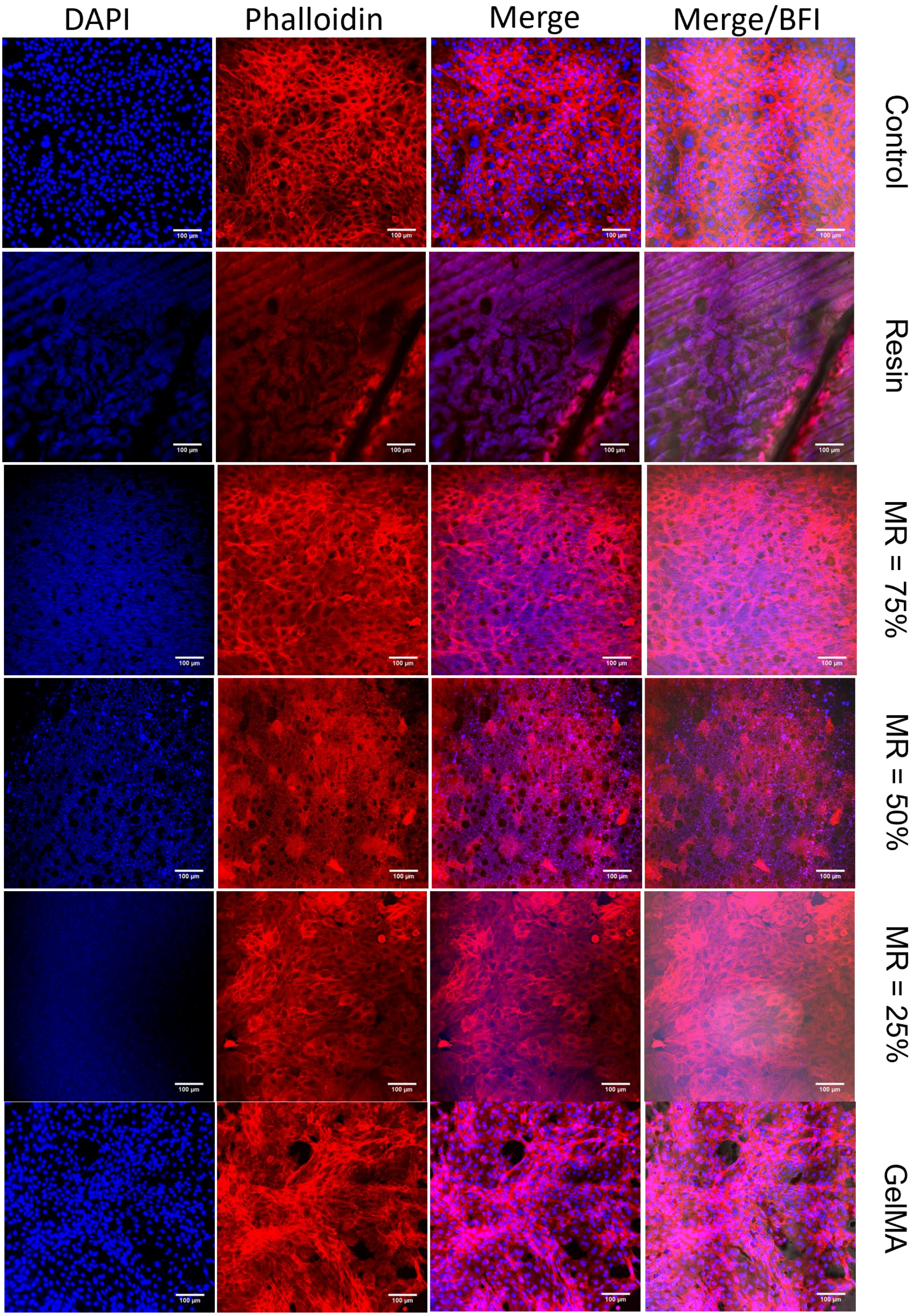

Supplementary Figure 18. Day 5 cell proliferation results for different mixture ratios including bright field images (BFI). Scale bar is 100 micron.

Metabolic activity study (AlamarBlue)

While the normalized metabolic activity data for different mixture ratios is presented in Figure 3 in the main text, here the non-normalized results are presented in Supplementary Figure 19. Supplementary Figure 20 presents statistical analysis on normalized values.

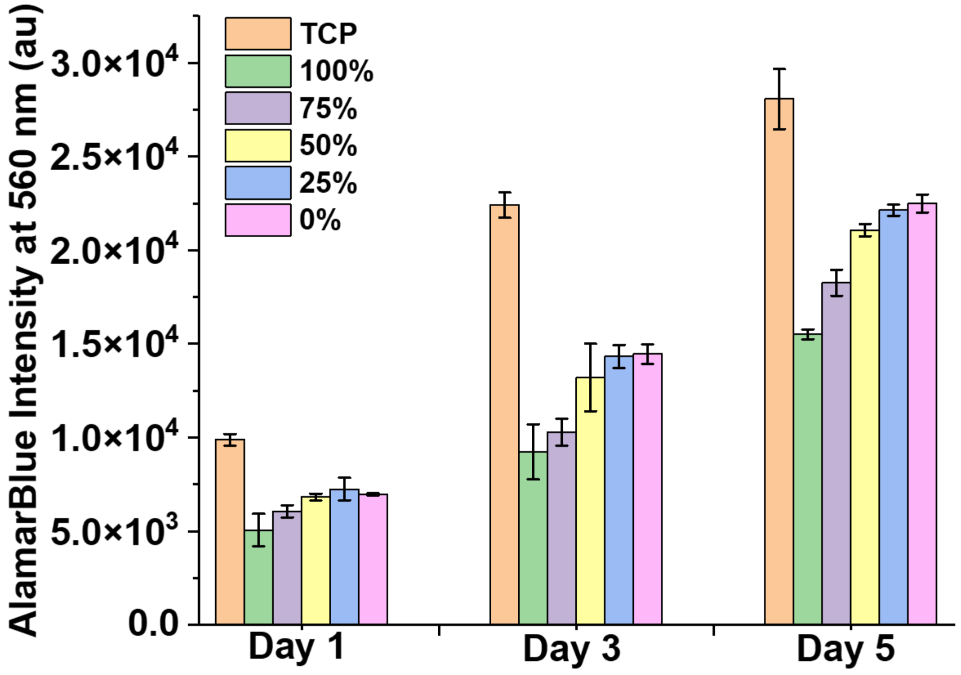

Supplementary Figure 19. Metabolic activity of the resin (100%), GelMA (0%), and composite samples with respect to the TCP on day 1, day 3, and day 5.

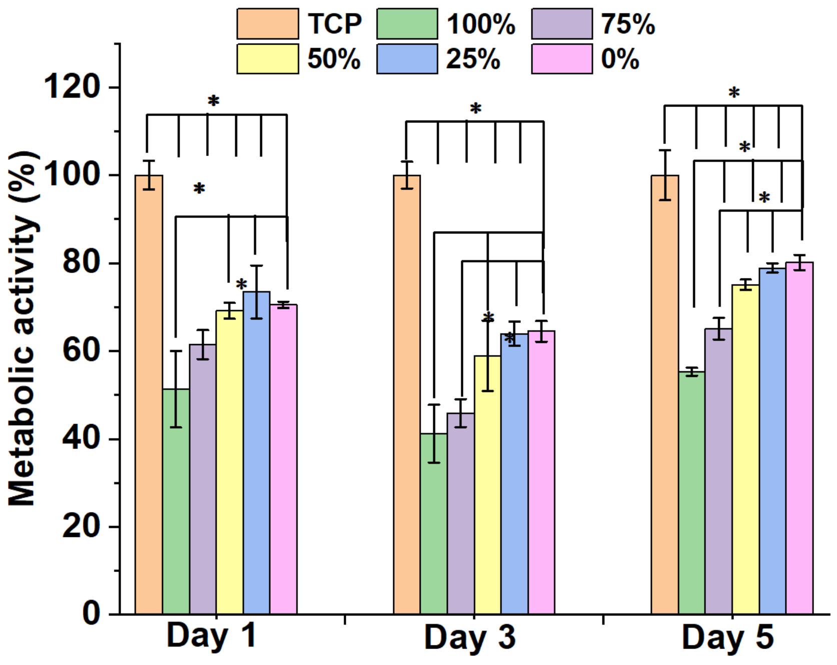

Supplementary Figure 20. Metabolic activity of cells cultured on samples with varying GelMA content [100% (resin), 75%, 50%, 25%, 0% (GelMA)] compared to TCP over 1, 3, and 5 days, assessed using the AlamarBlue assay. Higher GelMA content supports increased metabolic activity over time. Asterisks indicate statistically significant differences ($p < 0.05$).

Supplementary information 4: Chemical characterisation of the resin/GelMA composite

SI (4-1): Raman analysis

Raman analyses using (a) green laser (514 nm) and (b) IR laser (718 nm) over the full spectrum 100-3200 cm^{-1} are illustrated in Supplementary Figure 21. The power of the green laser for the liquid resin was set at 33% to avoid saturation, while the IR laser was emitted at 100% power.

In addition to the peaks discussed in the main text, there are other peaks between 1100 – 1450 cm^{-1} that exist in the liquid resin only, which are 1199, 1237, 1283 (shoulder peak), and 1408 cm^{-1}. The peaks at 1237 and 1283 cm^{-1} are within the range of C-O and C-C vibrations that present the epoxides and diminish after cure, hence, indicating the ring opening of epoxides and converting to ethers and polymerisation [1]. The bending vibrations of the CH2 group are present in the 1400-1450 cm^{-1} band, which correlates to the 1408 cm^{-1} peak as another indicator of acrylate reaction. The form of the Raman spectrum between 2700-3200 cm^{-1} where CH3 is strongly present, is similar to that described in literature for PEGs with mass of 1500 Da and above [2]; which is in agreement with the results from the MALDI analysis. The form of the graph between 2700-2950 cm^{-1} is similar for liquid and cured resin as this band belongs to the CH stretching vibrations.

a)

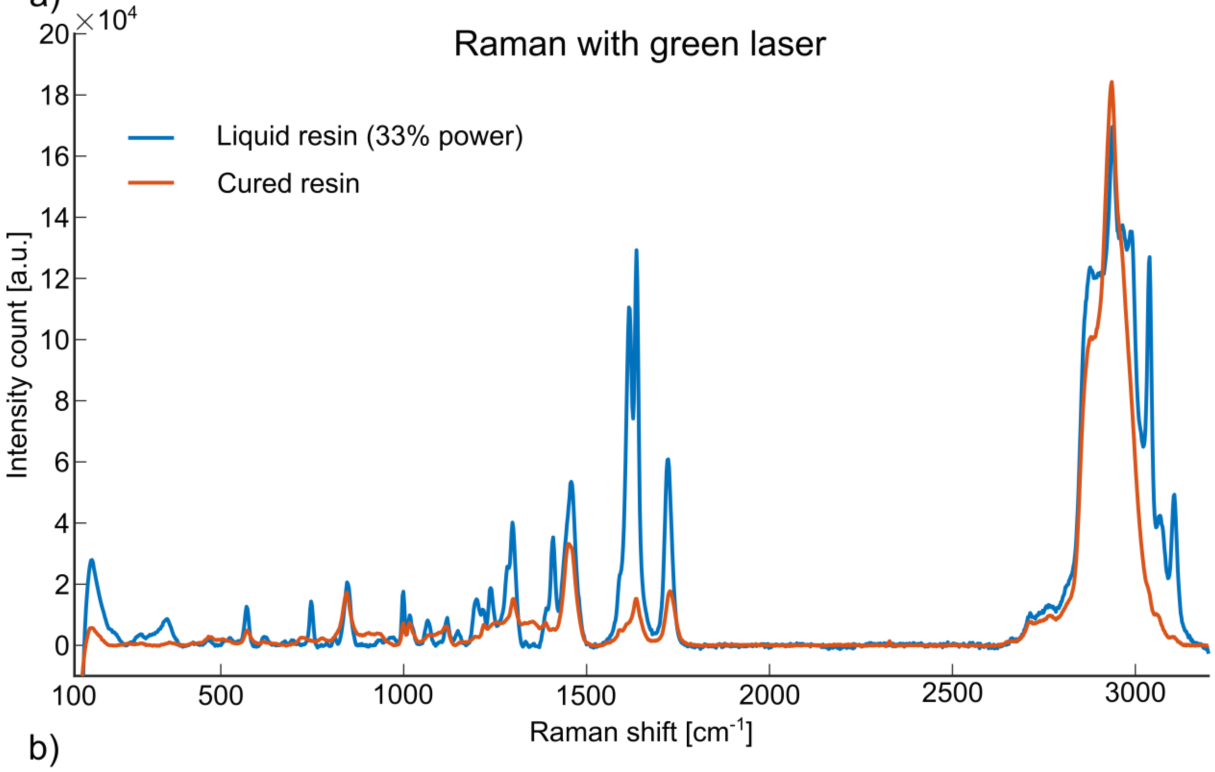

b)

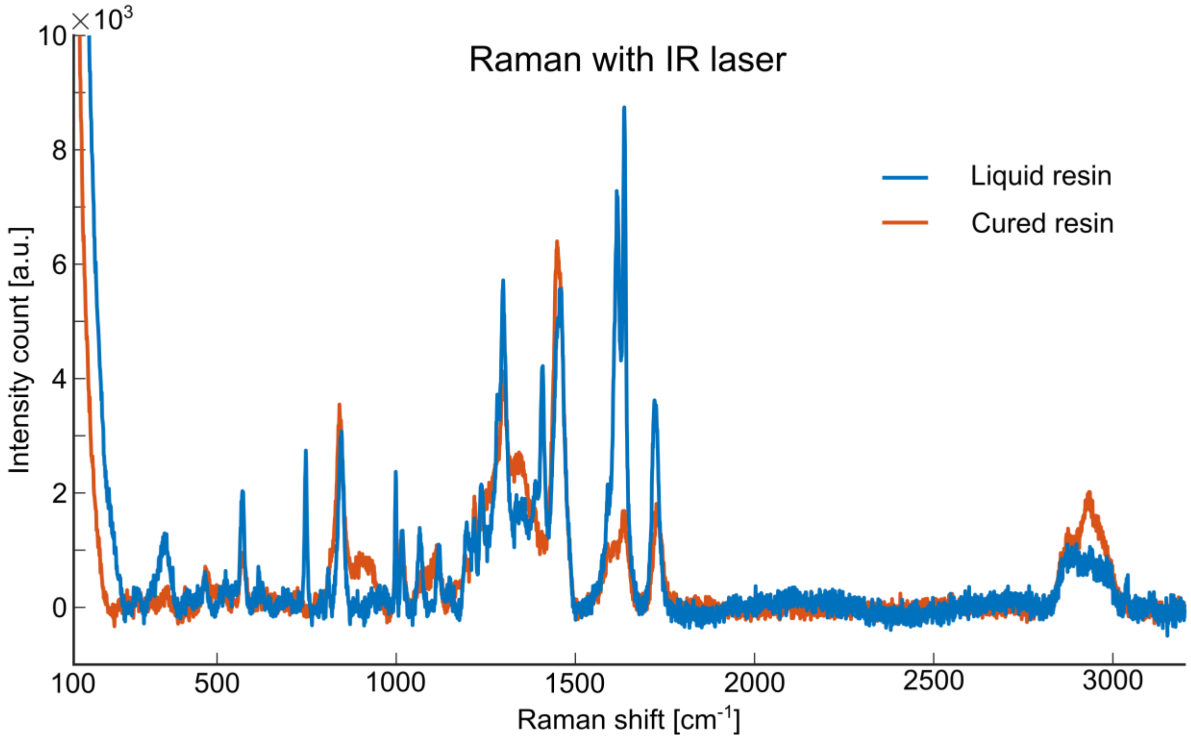

Supplementary Figure 21. Raman spectrum of the liquid resin vs. cured and washed resin using (a) green laser (514 nm), and (b) IR laser (718 nm).

SI (4-2): MALDI-TOF analysis

The mass of the PEG-based components with different number of Ethylene oxide ($M.Z^{-1} \approx 44$) is observed at different ranges for the liquid resin.

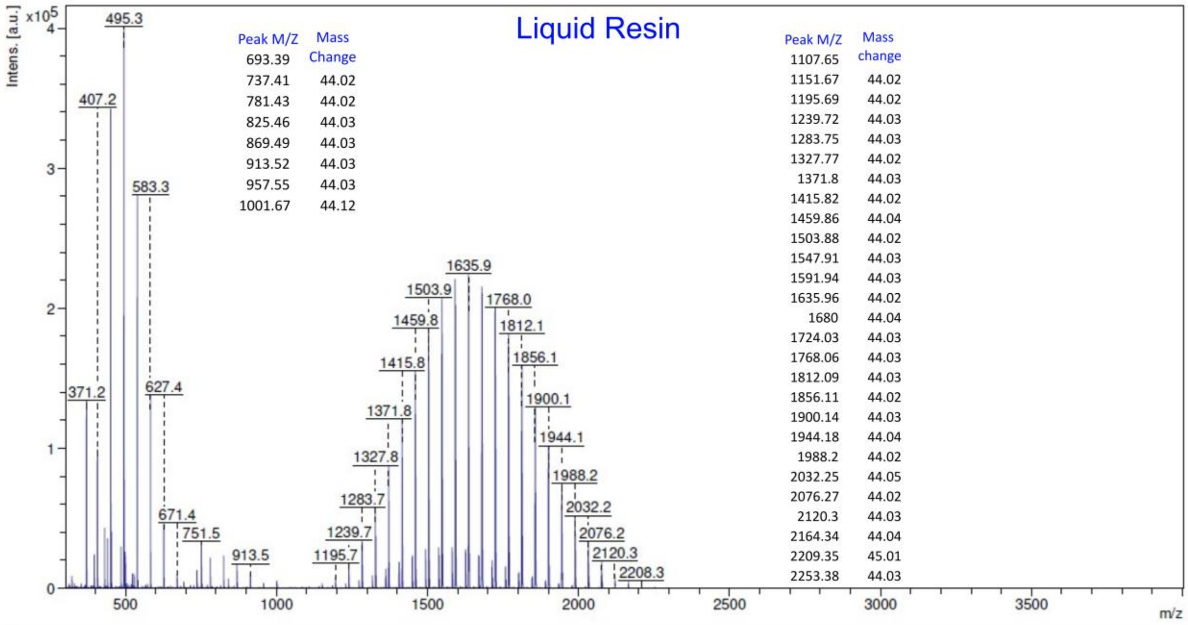

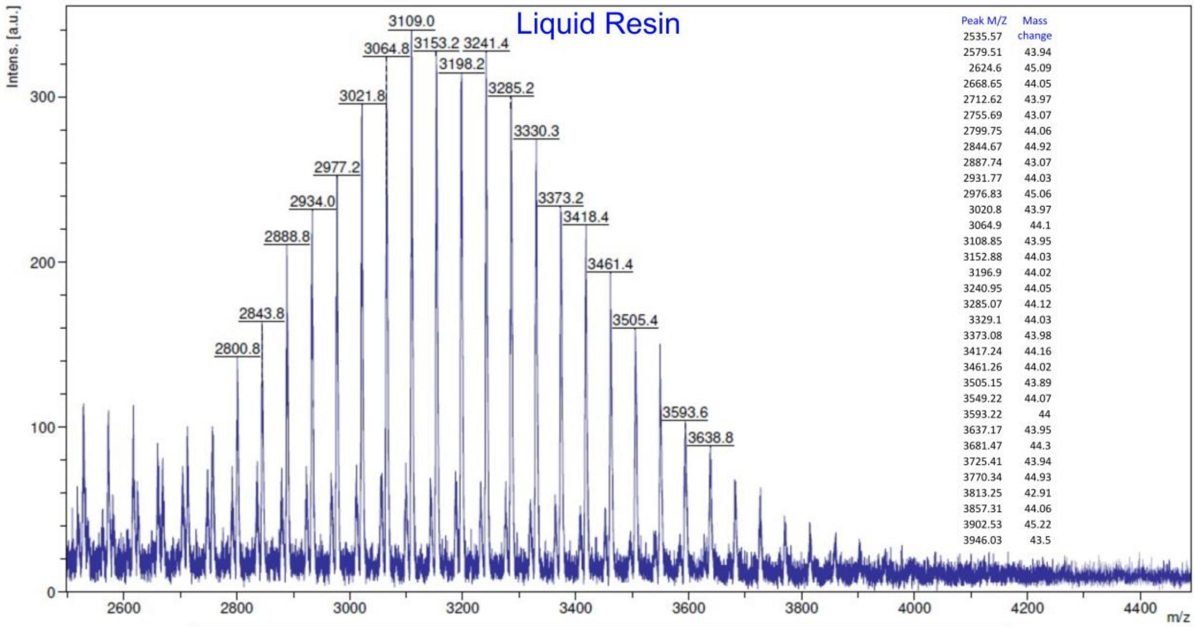

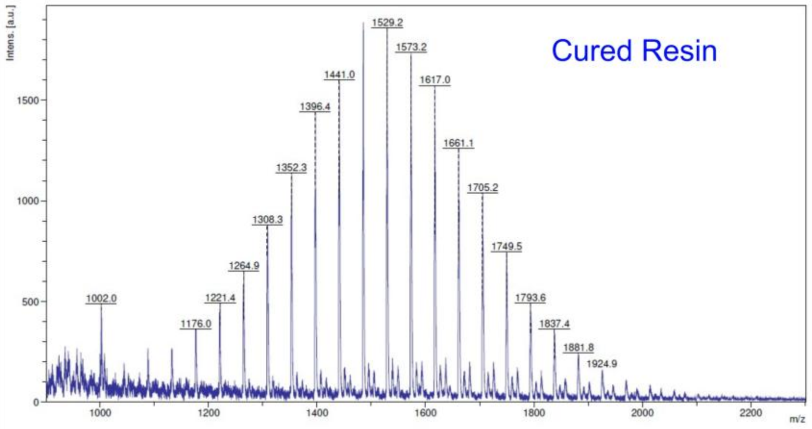

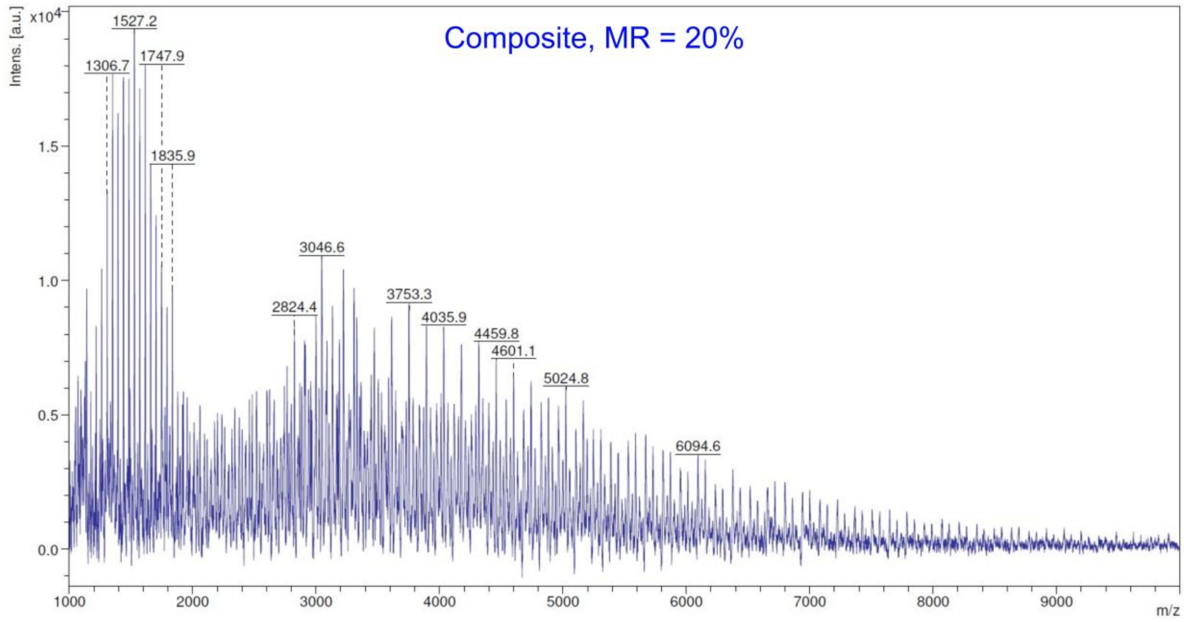
Composite, MR = 20%
Intens. [a.u.]
x10^4
1527.2
1306.7
1747.9
1835.9
3046.6
2824.4
3753.3
4035.9
4459.8
4601.1
5024.8
6094.6
m/z

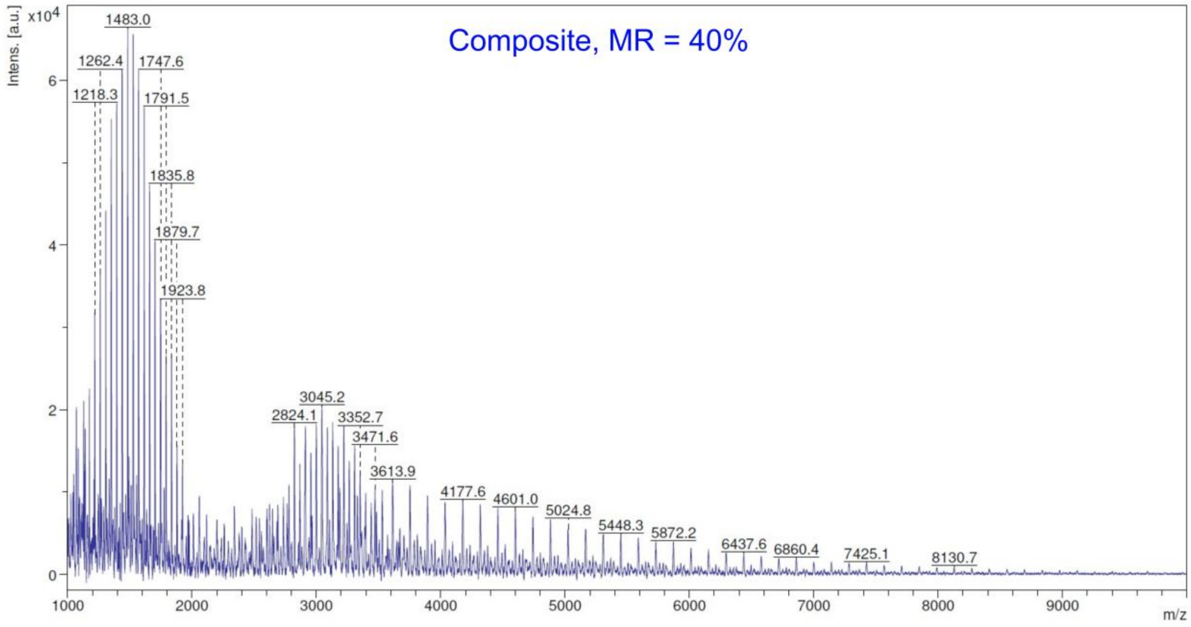
Composite, MR = 40%
Intens. [a.u.]
x10^4
1483.0
1262.4
1747.6
1218.3
1791.5
1835.8
1879.7
1923.8
3045.2
2824.1
3352.7
3471.6
3613.9
4177.6
4601.0
5024.8
5448.3
5872.2
6437.6
6860.4
7425.1
8130.7
m/z

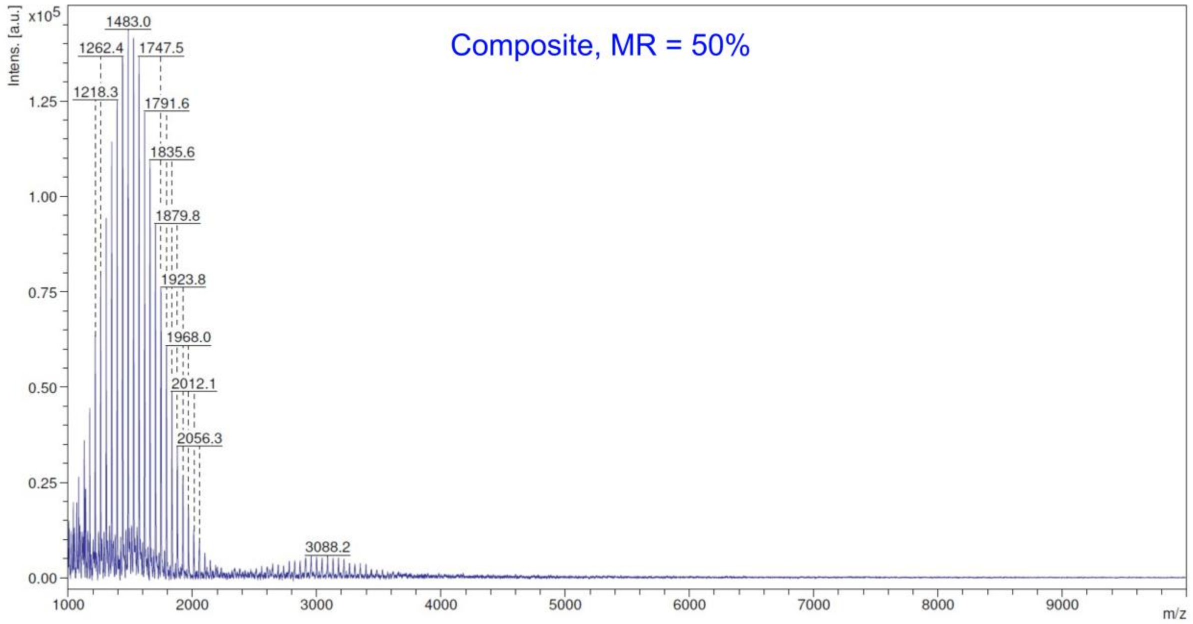
Composite, MR = 50%
Intens. [a.u.]
x10^5
1483.0
1262.4
1747.5
1218.3
1791.6
1835.6
1879.8
1923.8
1968.0
2012.1
2056.3
3088.2
m/z

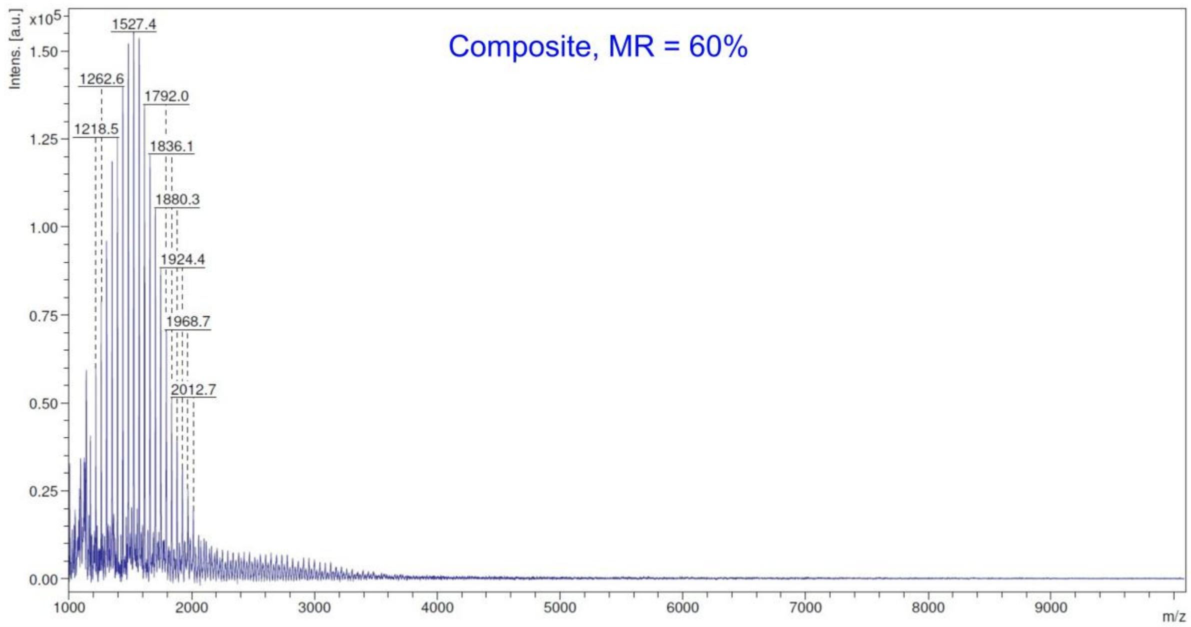

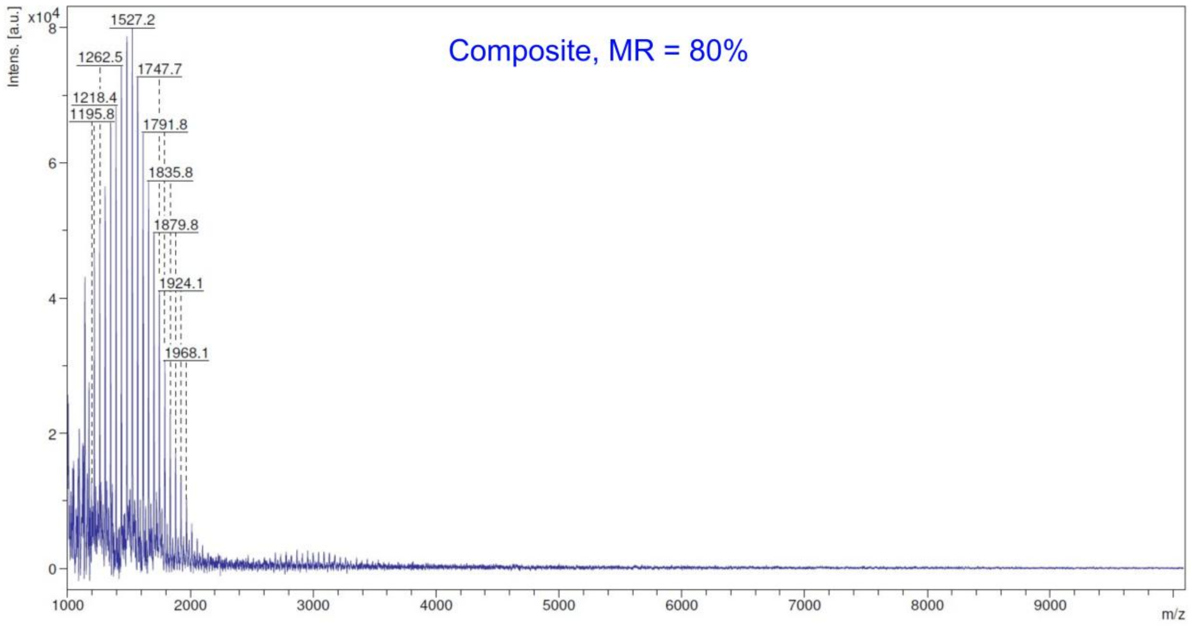

Supplementary Figure 22. MALDI-TOF results for the liquid resin, cured resin, and composites with different mixture ratios.

Supplementary information 5: Gradient manufacturing details

The gradient manufacturing setup and mixing tip are shown in Supplementary Figure 23.

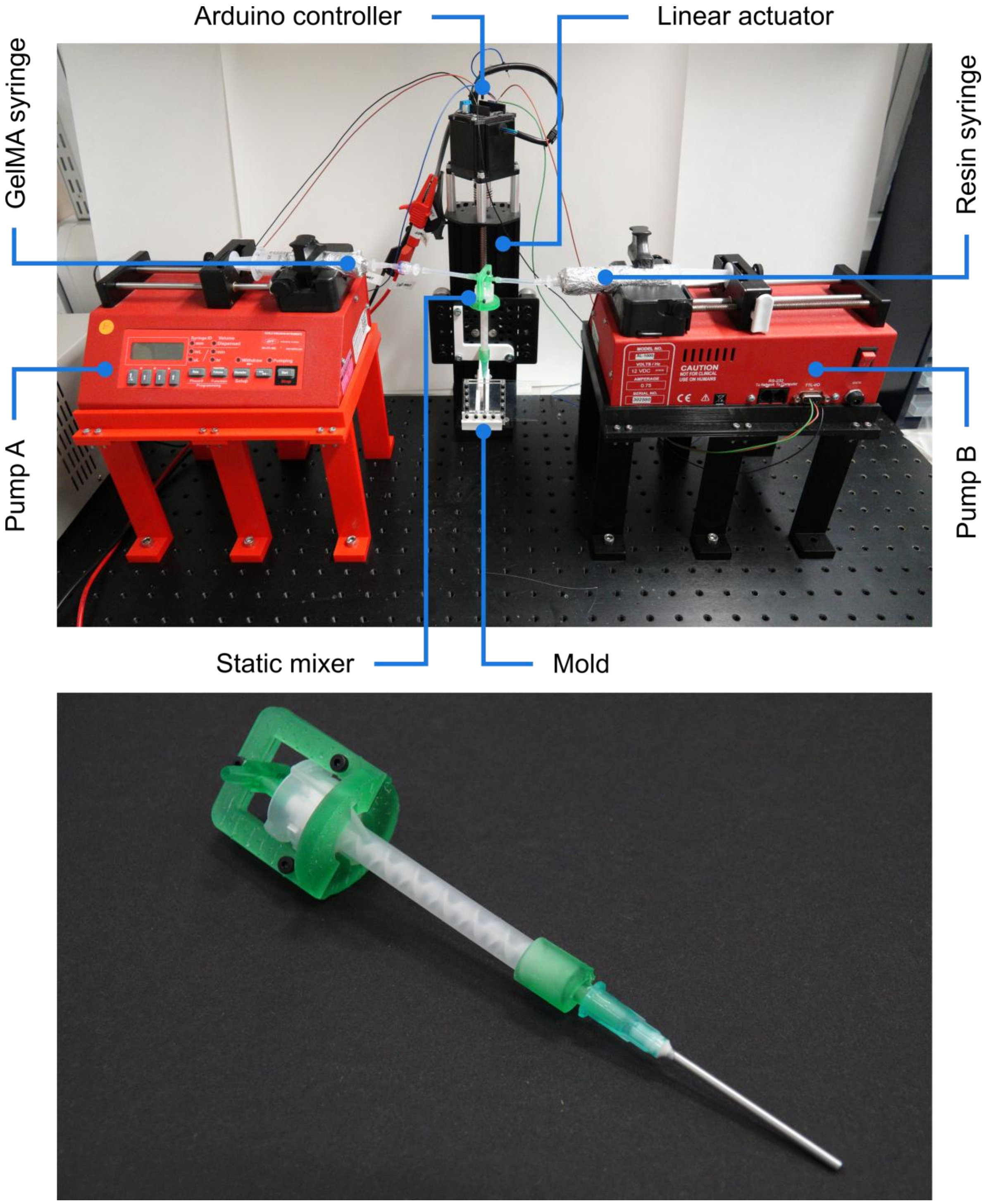

Supplementary Figure 23. Gradient sample manufacturing setup and the static mixer/nozzle.

Supplementary information 6: Gradient biocompatibility study

The surface of the gradient sample has very small local variations in height within microscale range. Therefore, sweeping of the focus was performed to capture the Live/Dead cells over a constant region. Corresponding bright field images (BFI) are presented as GIF images for different positions of gradient sample in the Supplementary Video 1.

The gradient of the color in the sample post culture as shown in Supplementary Figure 24, indicates the role of the mixture ratio and stiffness on the permeability and cell culture. Therefore, color cues can be also used to investigate and confirm gradient using image processing techniques.

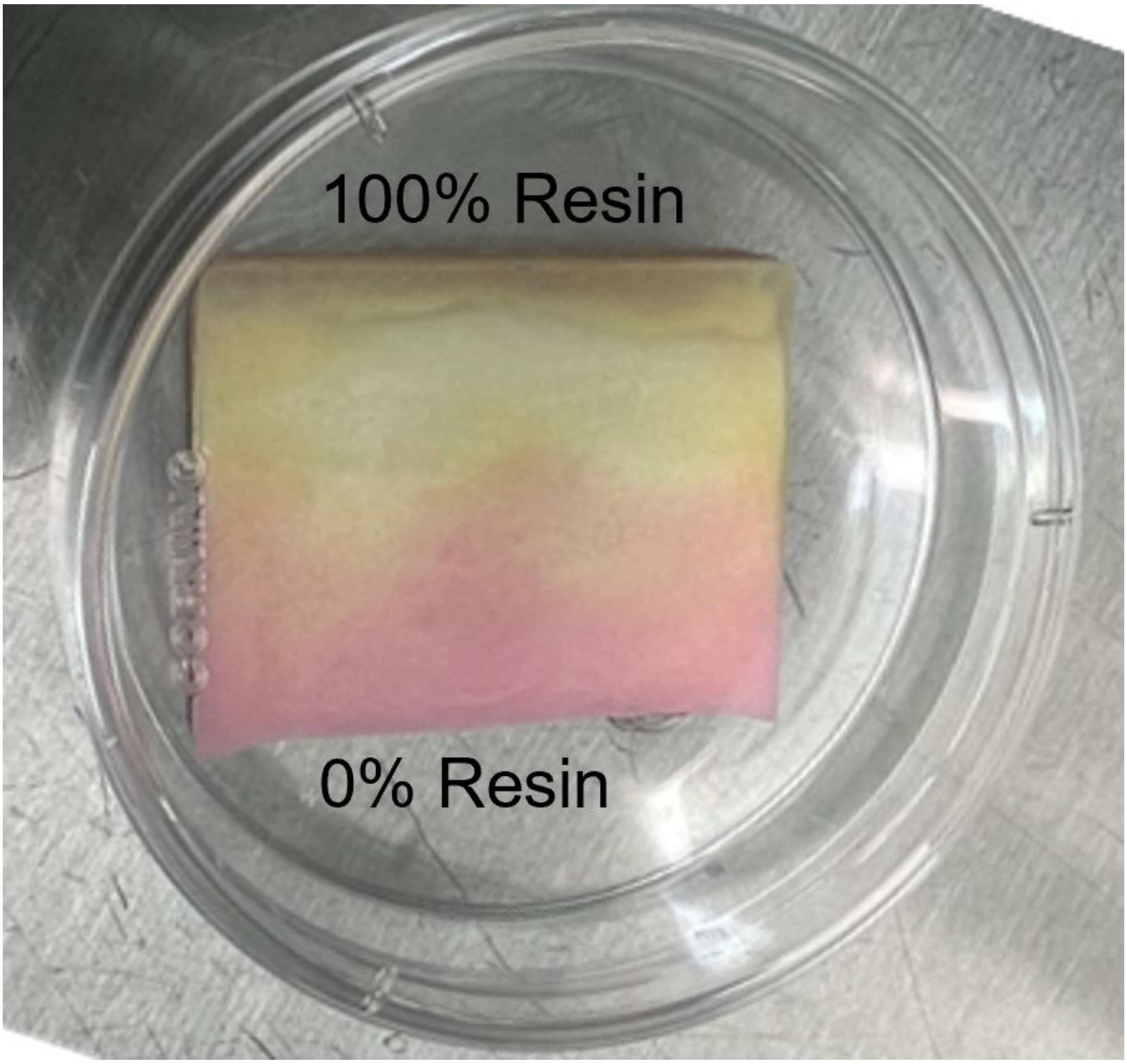

Supplementary Figure 24. Gradient sample after cell culture. The top side is resin-rich and the bottom side is GelMA-rich.

In addition to the mechanical measurements, the color change from yellow to white for a sample overexposed to light can be a cue for evaluating the gradient as shown in the Supplementary Figure 25a. This is due to the photobleaching by the fluorophore components in the resin that results in yellow color emission, however, it diminishes after some time as shown in the second photo (b) of another gradient sample.

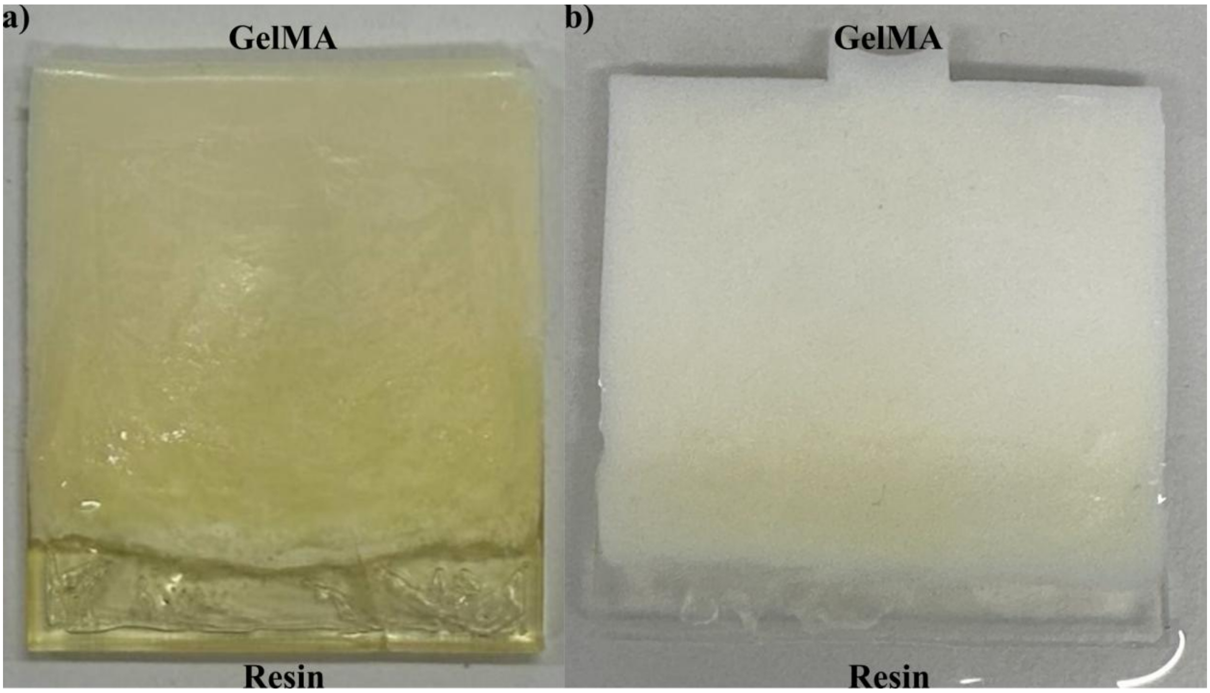

Supplementary Figure 25. a) Fluorophore yellow hue post manufacturing and b) diminished yellow color by time.

Supplementary information 7: Degradation and fatigue study

To study the degradation performance of the resin/GelMA composite, stiffness and hardness were measured on day 0, 7, and 14 post-manufacturing. Resin, GelMA, MR = 50% composite, and MR = 20% composite were considered for tensile tests and hardness measurements. Three tensile test specimens (n=3) for each mixture ratio on each test day were manufactured where 500 s of curing with 405 nm light was applied during manufacturing. Tensile test specimens followed the ISO 37 Type 2 geometry and hardness test specimens were disks of 20 mm diameter and 4 mm thickness. To ensure environmental conditions were representative of biological conditions, the specimens of day 7 and day 14 were stored in test tubes filled with PBS. In this regard, they were removed from the mold on days zero and transferred to the test tubes, which were floating in a paraffin oil bath on a hot plate that maintained the temperature at 37-38 degrees.

To perform tensile tests under physiologically relevant humidity conditions, a custom test chamber was designed and manufactured specifically for tensile testing in de-ionized water (DI water), as shown in Supplementary Figure 25, where a composite specimen (MR = 20%) is loaded. Since chamber is filled with water and the load capacity of soft samples such as GelMA and MR = 20% are low, the buoyancy force of the top moving gripper is first measured without testing a specimen. Subsequently, the buoyancy force is subtracted from the specimen's force-displacement data to obtain the net force of elastic deformation.

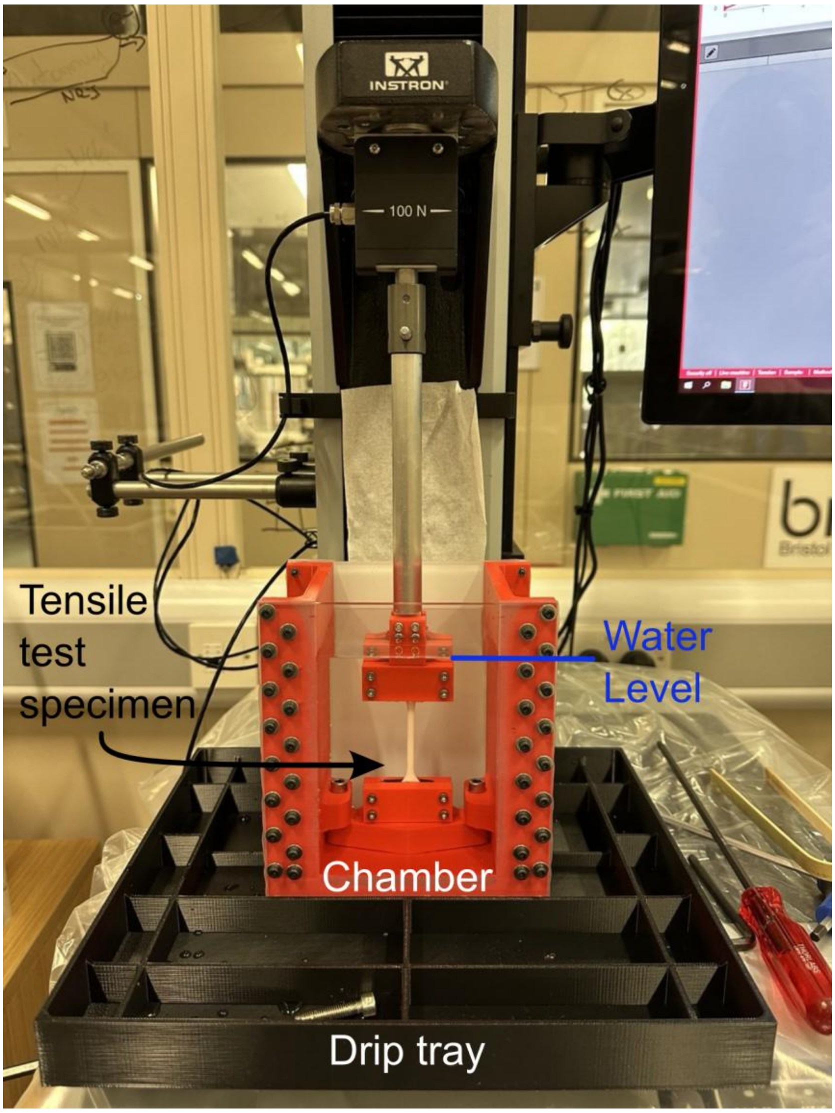

Supplementary Figure 26. Test chamber designed for tensile test in DI water.

On day 0, all samples were removed from the molds. For day 0 analysis, tensile tests were performed immediately after removing specimens from molds. For day 7 and day 14 tests, specimens were stored in PBS in test tubes from day 0. The variation of elastic modulus for the resin, GelMA, MR = 20%, and MR = 50% composite samples are shown in Supplementary Figure 27 (a-d). For the ease of evaluation, the elastic modulus of the samples are normalized with respect to the modulus on day 0. For all samples except MR = 50%, each data point and error bar represent the mean value and standard deviation in modulus of three specimens. The samples of MR = 50% were mishandled, resulting in two samples having large difference in elastic modulus.

Overall, the results indicate that the pure resin degrades quickly in the liquid environment, with an additional data point on day 1 reveals that the modulus is reduced by half after 24 hours in PBS at 37-38 degrees (Supplementary Figure 27a). However, the rate of degradation slows down after the first day, with the change in elastic modulus between day 7 and 14 being smaller than the change observed between day 1 and 7. A similar trend was observed in GelMA, where the elastic modulus on day 14 is about 20% of its initial value on day 0. In contrast to pure resin and pure GelMA, the composite degrades much more slowly. For example, the mean elastic modulus of MR = 20% composite decreased by less than 2% over 14 days in similar condition to other samples. A similar trend is also visible for the mean value of the modulus of MR = 50% composite.

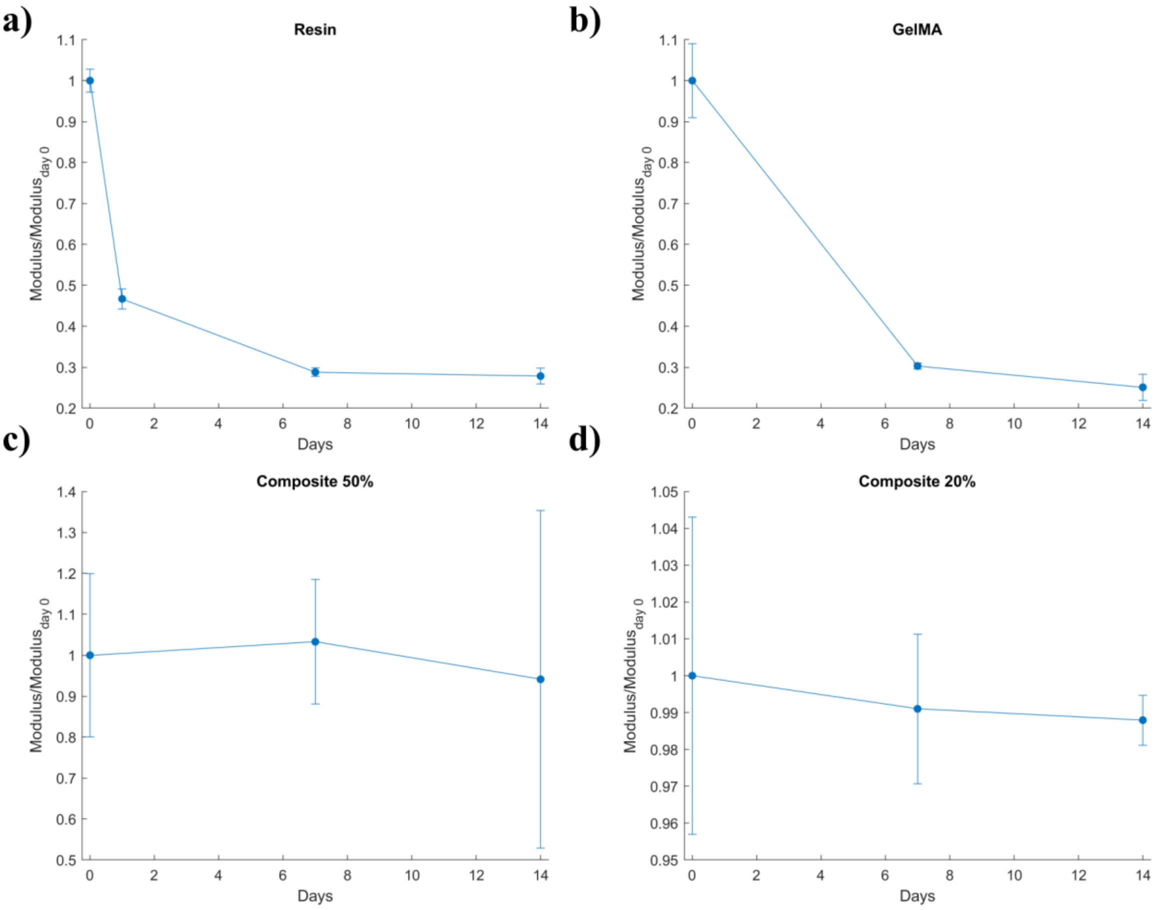

Supplementary Figure 27. Variation of elastic modulus over 14 days with respect to day 0 for a) resin, b) GelMA, c) MR = 50% and d) MR = 20%.

In addition to the tensile testing, the variation of hardness over 14 days was also assessed using durometry as shown in Supplementary Figure 28 (a-b). Both absolute and normalized values of hardness for resin (Shore A) and composites of MR = 20% and 50% (Shore 00) are reported.

a)

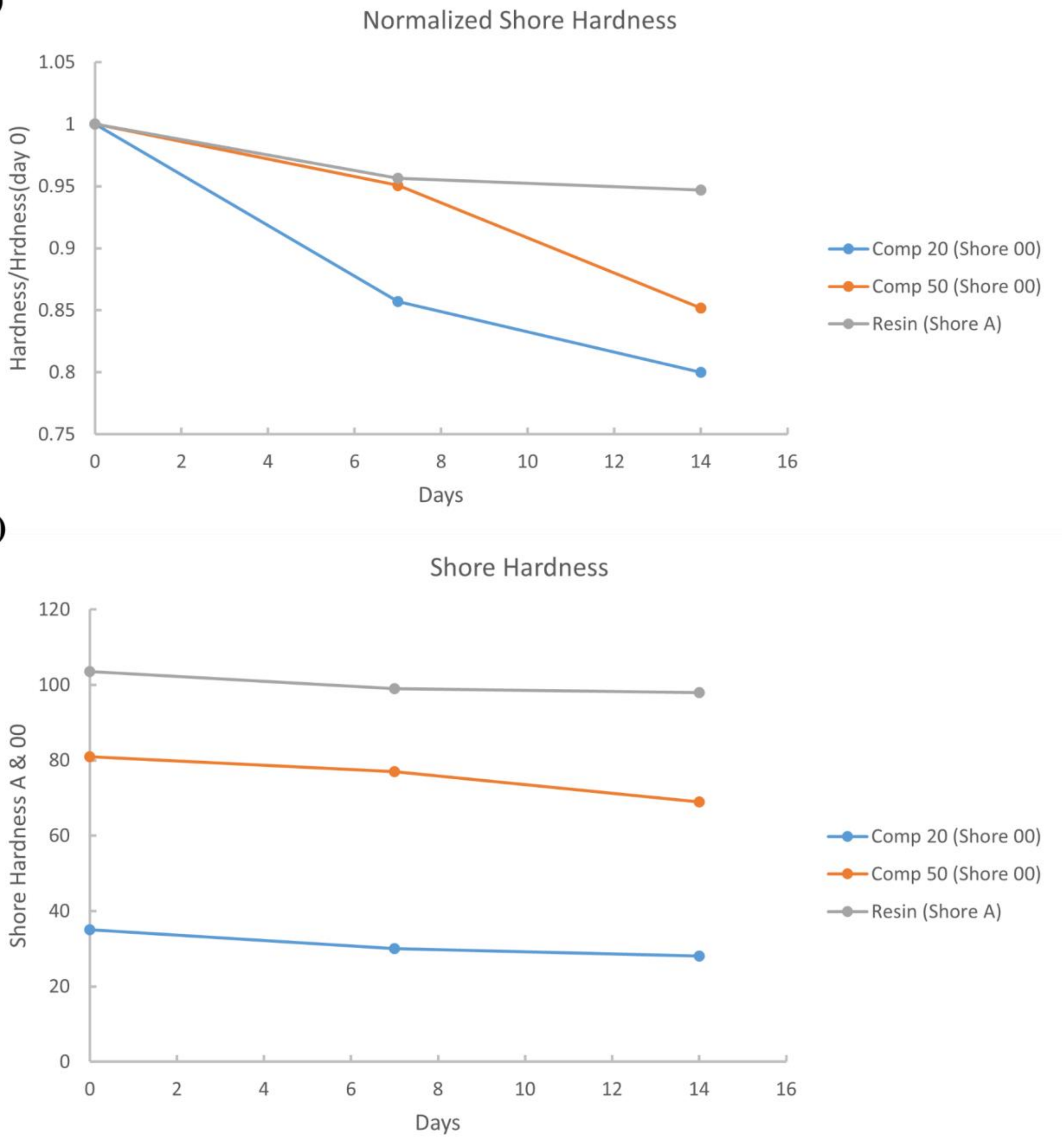

Supplementary Figure 28. Variation of hardness over 14 days period in PBS and about 37°C for resin, MR = 20% and MR = 50%: a) normalized hardness with respect to day 0, and b) absolute hardness values.

As resin is the harder component of this composite material and is more brittle, we study the fatigue behavior of the resin specimen and the fatigue behavior of GelMA is also studied in the literature [3,4]. Fatigue analysis is based on the interpretation of data from low-speed quasi-static cyclic tensile tests performed in DI-water. Resin tensile test specimen with ISO 37 type 2 geometry was 3D-printed and tests were performed in the chamber shown in Supplementary Figure 26. The resin specimen was maintained dry before entering the chamber and the total test takes about 65 hours. The test speed was set at 2 $mm.min^{-1}$ and the cycles were performed at maximum 30 N force. The full cyclic force-displacement data is presented in Supplementary Figure 29a where significant creep above 1.4 mm (5.6 % strain) is observed in the specimen. Individual force-displacement diagrams are isolated and illustrated in Supplementary Figure 29b for cycle 1, 5000, 10000 and 15000. In this figure two trends are visible, reduction of stiffness by increasing the cycle number and time and increase in the area between loading and unloading cycle.

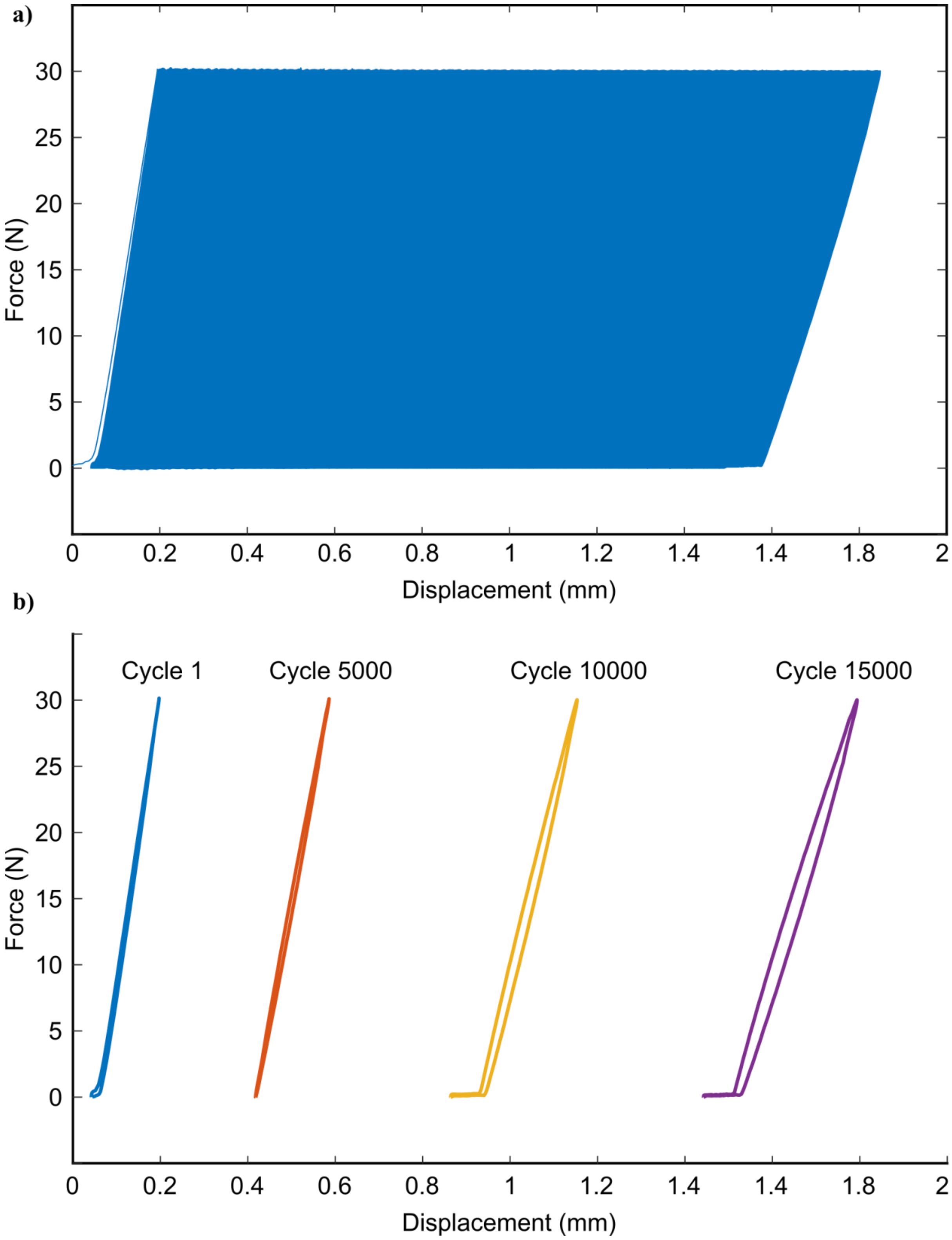

Supplementary Figure 29. Force-displacement diagrams of quasi-static cyclic tests in DI-water for a resin sample (MR = 100%), a) all 16,000 cycles, and b) diagrams of cycle 1, 5,000, 10,000 and 15,000.

Previously, as shown in Supplementary Figure 27, the reducing trend in the modulus of the resin stored in PBS was observed, where significant reduction in modulus was observed just one day after soaking. In the quasi-static cyclic tests, the time to reach to 15,000th cycle is about 61 hours, and the results confirm the rapid reduction of stiffness in aquatic environment as shown in Supplementary Figure 30a. The area between the loading and unloading cycle is a measure of dissipation and damping of the

material, where a continuous incremental trend by cycle number and time in water is observed as shown in Supplementary Figure 30b.

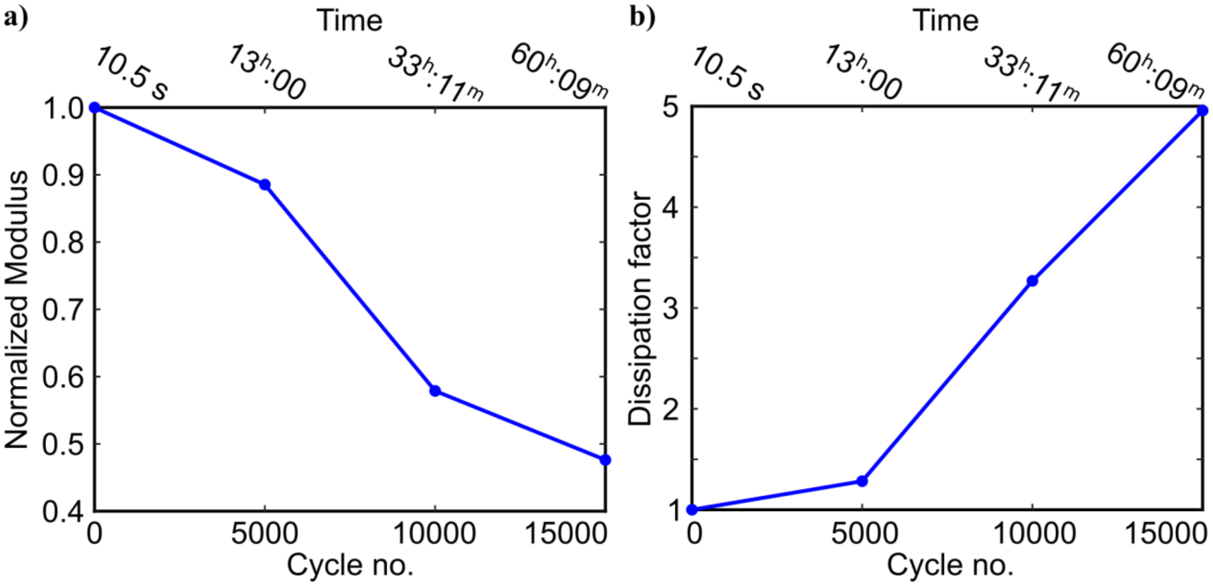

Supplementary Figure 30. Effect of cycle number and time in DI-water on the resin sample is shown for a) the modulus normalized to the modulus of the first cycle, and b) the dissipation with respect to the dissipation of the first cycle.

References

[1] Kuzmin, V. et al. Raman spectra of polyethylene glycols: Comparative experimental and dft study. J. Mol. Struct. 1217, 128331 (2020).

[2] Miranda, A. M. et al. Line shape analysis of the raman spectra from pure and mixed biofuels esters compounds. Fuel 115, 118–125 (2014).

[3] Liang, Q., et al. A Stretching Force Control-Based Cyclic Loading Method for the Evaluation of Mechanical Properties of Gelation Methacrylate (GelMA) Microfibers. Micromachines 13.10 (2022): 1703.

[4] Wang, M., et al. Soft, strong, tough, and durable protein-based fiber hydrogels. Proceedings of the National Academy of Sciences 120.8 (2023): e2213030120.
