# Supplementary material for: Hybrid diacrylate resin-gelatin methacryloyl composite with bone-to-brain stiffness range
Source: Commun Mater. 2025 Oct 2;6(1):219. doi: 10.1038/s43246-025-00931-y (PMC12542801; doi:10.1038/s43246-025-00931-y)
Supplement: Supplementary file 2 — Description of Additional Supplementary File [file 43246_2025_931_MOESM2_ESM.pdf]

Description of additional supplementary data

Supplementary Video 1:

The cell viability analysis of the gradient sample required a sweep of optical focus due to surface roughness. To capture cell viability data in presence of this surface height variation, GIF images are created by sweeping the focus presented in this supplementary video. The images are taken at different positions along the length of the gradient sample representing different stiffnesses and mixture ratios.
